# International genome-wide meta-analysis identifies new primary biliary cirrhosis risk loci and targetable pathogenic pathways

**DOI:** 10.1038/ncomms9019

**Published:** 2015-09-22

**Authors:** Heather J. Cordell, Younghun Han, George F. Mells, Yafang Li, Gideon M. Hirschfield, Casey S. Greene, Gang Xie, Brian D. Juran, Dakai Zhu, David C. Qian, James A. B. Floyd, Katherine I. Morley, Daniele Prati, Ana Lleo, Daniele Cusi, Canadian-US PBC Consortium, Erik M Schlicht, Craig Lammert, Elizabeth J Atkinson, Landon L Chan, Mariza de Andrade, Tobias Balschun, Andrew L Mason, Robert P Myers, Jinyi Zhang, Piotr Milkiewicz, Jia Qu, Joseph A Odin, Velimir A Luketic, Bruce R Bacon, Henry C Bodenheimer Jr, Valentina Liakina, Catherine Vincent, Cynthia Levy, Peter K Gregersen, Italian PBC Genetics Study Group, Piero L Almasio, Domenico Alvaro, Pietro Andreone, Angelo Andriulli, Cristina Barlassina, Pier Maria Battezzati, Antonio Benedetti, Francesca Bernuzzi, Ilaria Bianchi, Maria Consiglia Bragazzi, Maurizia Brunetto, Savino Bruno, Giovanni Casella, Barbara Coco, Agostino Colli, Massimo Colombo, Silvia Colombo, Carmela Cursaro, Lory Saveria Crocè, Andrea Crosignani, Maria Francesca Donato, Gianfranco Elia, Luca Fabris, Carlo Ferrari, Annarosa Floreani, Barbara Foglieni, Rosanna Fontana, Andrea Galli, Roberta Lazzari, Fabio Macaluso, Federica Malinverno, Fabio Marra, Marco Marzioni, Alberto Mattalia, Renzo Montanari, Lorenzo Morini, Filomena Morisco, Mousa Hani S, Luigi Muratori, Paolo Muratori, Grazia A Niro, Vincenzo O Palmieri, Antonio Picciotto, Mauro Podda, Piero Portincasa, Vincenzo Ronca, Floriano Rosina, Sonia Rossi, Ilaria Sogno, Giancarlo Spinzi, Marta Spreafico, Mario Strazzabosco, Sonia Tarallo, Mirko Tarocchi, Claudio Tiribelli, Pierluigi Toniutto, Maria Vinci, Massimo Zuin, UK-PBC Consortium, Chin Lye Ch'ng, Mesbah Rahman, Tom Yapp, Richard Sturgess, Christopher Healey, Marek Czajkowski, Anton Gunasekera, Pranab Gyawali, Purushothaman Premchand, Kapil Kapur, Richard Marley, Graham Foster, Alan Watson, Aruna Dias, Javaid Subhani, Rory Harvey, Roger McCorry, David Ramanaden, Jaber Gasem, Richard Evans, Thiriloganathan Mathialahan, Christopher Shorrock, George Lipscomb, Paul Southern, Jeremy Tibble, David Gorard, Altaf Palegwala, Susan Jones, Marco Carbone, Mohamed Dawwas, Graeme Alexander, Sunil Dolwani, Martin Prince, Matthew Foxton, David Elphick, Harriet Mitchison, Ian Gooding, Mazn Karmo, Sushma Saksena, Mike Mendall, Minesh Patel, Roland Ede, Andrew Austin, Joanna Sayer, Lorraine Hankey, Christopher Hovell, Neil Fisher, Martyn Carter, Konrad Koss, Andrzej Piotrowicz, Charles Grimley, David Neal, Guan Lim, Sass Levi, Aftab Ala, Andrea Broad, Athar Saeed, Gordon Wood, Jonathan Brown, Mark Wilkinson, Harriet Gordon, John Ramage, Jo Ridpath, Theodore Ngatchu, Bob Grover, Syed Shaukat, Ray Shidrawi, George Abouda, Faiz Ali, Ian Rees, Imroz Salam, Mark Narain, Ashley Brown, Simon Taylor-Robinson, Simon Williams, Leonie Grellier, Paul Banim, Debashis Das, Andrew Chilton, Michael Heneghan, Howard Curtis, Markus Gess, Ian Drake, Mark Aldersley, Mervyn Davies, Rebecca Jones, Alastair McNair, Raj Srirajaskanthan, Maxton Pitcher, Sambit Sen, George Bird, Adrian Barnardo, Paul Kitchen, Kevin Yoong, Oza Chirag, Nurani Sivaramakrishnan, George MacFaul, David Jones, Amir Shah, Chris Evans, Subrata Saha, Katharine Pollock, Peter Bramley, Ashis Mukhopadhya, Andrew Fraser, Peter Mills, Christopher Shallcross, Stewart Campbell, Andrew Bathgate, Alan Shepherd, John Dillon, Simon Rushbrook, Robert Przemioslo, Christopher Macdonald, Jane Metcalf, Udi Shmueli, Andrew Davis, Asifabbas Naqvi, Tom Lee, Stephen D Ryder, Jane Collier, Howard Klass, Mary Ninkovic, Matthew Cramp, Nicholas Sharer, Richard Aspinall, Patrick Goggin, Deb Ghosh, Andrew Douds, Barbara Hoeroldt, Jonathan Booth, Earl Williams, Hyder Hussaini, William Stableforth, Reuben Ayres, Douglas Thorburn, Eileen Marshall, Andrew Burroughs, Steven Mann, Martin Lombard, Paul Richardson, Imran Patanwala, Julia Maltby, Matthew Brookes, Ray Mathew, Samir Vyas, Saket Singhal, Dermot Gleeson, Sharat Misra, Jeff Butterworth, Keith George, Tim Harding, Andrew Douglass, Simon Panter, Jeremy Shearman, Gary Bray, Graham Butcher, Daniel Forton, John Mclindon, Matthew Cowan, Gregory Whatley, Aditya Mandal, Hemant Gupta, Pradeep Sanghi, Sanjiv Jain, Steve Pereira, Geeta Prasad, Gill Watts, Mark Wright, James Neuberger, Fiona Gordon, Esther Unitt, Allister Grant, Toby Delahooke, Andrew Higham, Alison Brind, Mark Cox, Subramaniam Ramakrishnan, Alistair King, Carole Collins, Simon Whalley, Andy Li, Jocelyn Fraser, Andrew Bell, Voi Shim Wong, Amit Singhal, Ian Gee, Yeng Ang, Rupert Ransford, James Gotto, Charles Millson, Jane Bowles, Caradog Thomas, Melanie Harrison, Roman Galaska, Jennie Kendall, Jessica Whiteman, Caroline Lawlor, Catherine Gray, Keith Elliott, Caroline Mulvaney-Jones, Lucie Hobson, Greta Van Duyvenvoorde, Alison Loftus, Katie Seward, Ruth Penn, Jane Maiden, Rose Damant, Janeane Hails, Rebecca Cloudsdale, Valeria Silvestre, Sue Glenn, Eleanor Dungca, Natalie Wheatley, Helen Doyle, Melanie Kent, Caroline Hamilton, Delyth Braim, Helen Wooldridge, Rachel Abrahams, Alison Paton, Nicola Lancaster, Andrew Gibbins, Karen Hogben, Phillipa Desousa, Florin Muscariu, Janine Musselwhite, Alexandra McKay, LaiTing Tan, Carole Foale, Jacqueline Brighton, Kerry Flahive, Estelle Nambela, Paula Townshend, Chris Ford, Sophie Holder, Caroline Palmer, James Featherstone, Mariam Nasseri, Joy Sadeghian, Bronwen Williams, Carol Thomas, Sally-Ann Rolls, Abigail Hynes, Claire Duggan, Sarah Jones, Mary Crossey, Glynis Stansfield, Carolyn MacNicol, Joy Wilkins, Elva Wilhelmsen, Parizade Raymode, Hye-Jeong Lee, Emma Durant, Rebecca Bishop, Noma Ncube, Sherill Tripoli, Rebecca Casey, Caroline Cowley, Richard Miller, Kathryn Houghton, Samantha Ducker, Fiona Wright, Bridget Bird, Gwen Baxter, Janie Keggans, Maggie Hughes, Emma Grieve, Karin Young, D Williams, Kate Ocker, Frances Hines, Kirsty Martin, Caron Innes, Talal Valliani, Helen Fairlamb, Sarah Thornthwaite, Anne Eastick, Elizabeth Tanqueray, Jennifer Morrison, Becky Holbrook, Julie Browning, Kirsten Walker, Susan Congreave, Juliette Verheyden, Susan Slininger, Lizzie Stafford, Denise O'Donnell, Mark Ainsworth, Susan Lord, Linda Kent, Linda March, Christine Dickson, Diane Simpson, Beverley Longhurst, Maria Hayes, Ervin Shpuza, Nikki White, Sarah Besley, Sallyanne Pearson, Alice Wright, Linda Jones, Emma Gunter, Hannah Dewhurst, Anna Fouracres, Liz Farrington, Lyn Graves, Suzie Marriott, Marina Leoni, David Tyrer, Kate Martin, Lola Dali-kemmery, Victoria Lambourne, Marie Green, Dawn Sirdefield, Kelly Amor, Julie Colley, Bal Shinder, Jayne Jones, Marisa Mills, Mandy Carnahan, Natalie Taylor, Kerenza Boulton, Julie Tregonning, Carly Brown, Gayle Clifford, Emily Archer, Maria Hamilton, Janette Curtis, Tracey Shewan, Sue Walsh, Karen Warner, Kimberley Netherton, Mcdonald Mupudzi, Bridget Gunson, Jane Gitahi, Denise Gocher, Sally Batham, Hilary Pateman, Senayon Desmennu, Jill Conder, Darren Clement, Susan Gallagher, Jacky Orpe, PuiChing Chan, Lynn Currie, Lynn O'Donohoe, Metod Oblak, Lisa Morgan, Marie Quinn, Isobel Amey, Yolanda Baird, Donna Cotterill, Lourdes Cumlat, Louise Winter, Sandra Greer, Katie Spurdle, Joanna Allison, Simon Dyer, Helen Sweeting, Jean Kordula, M. Eric Gershwin, Carl A. Anderson, Konstantinos N. Lazaridis, Pietro Invernizzi, Michael F. Seldin, Richard N. Sandford, Christopher I. Amos, Katherine A. Siminovitch

**Affiliations:** 1 Institute of Genetic Medicine, Newcastle University, Newcastle upon Tyne NE1 3BZ, UK; 2 Center for Genomic Medicine, Department of Community and Family Medicine, Geisel School of Medicine, Dartmouth College, Lebanon, New Hampshire 03755, USA; 3 Academic Department of Medical Genetics, Cambridge University, Cambridge CB2 0QQ, UK; 4 Centre for Liver Research and NIHR Biomedical Research Unit, University of Birmingham, Birmingham B15 2TT, UK; 5 Department of Genetics, Institute for Quantitative Biomedical Sciences, Geisel School of Medicine, Dartmouth College, Hanover, New Hampshire 03755, USA; 6 Mount Sinai Hospital, Lunenfeld-Tanenbaum Research Institute and Toronto General Research Institute, Toronto, Ontario M5G 1X5, Canada; 7 Center for Basic Research in Digestive Diseases, Mayo Clinic, Rochester, Minnesota 55905, USA; 8 Wellcome Trust Sanger Institute, Wellcome Trust Genome Campus, Hinxton CB10 1SA, UK; 9 William Harvey Research Institute, Queen Mary University of London, London EC1M 6BQ, UK; 10 Institute of Psychiatry, Psychology & Neuroscience, Kings College London, London SE5 8AF, UK; 11 Department of Transfusion Medicine and Hematology, Ospedale Alessandro Manzoni, Lecco 23900, Italy; 12 Center for Autoimmune Liver Diseases, IRCCS Instituto Clinico Humanitas, 20089 Rozzano, Italy; 13 Università degli Studi di Milano, Milan 20129, Italy; 14 Istituto di Tecnologie Biomediche Consiglio Nazionale delle Ricerche, Via Fratelli Cervi, 93, 20090 Segrate, Milan, Italy; 15 University of California – Davis, Davis, California 95616, USA; 16 Department of Medicine, University of Toronto, Toronto, Ontario, Canada M5G 1X5; 17 Department of Immunology, University of Toronto, Toronto, Ontario, Canada M5G 1X5; 18 Department of Molecular Genetics, University of Toronto, Toronto, Ontario, Canada M5G 1X5; 19 Division of Biomedical Statistics and Informatics, Mayo Clinic, Rochester, MN 55905, USA; 20 Institute of Clinical Molecular Biology, Christian-Albrechts-University of Kiel, 24118, Kiel Germany; 21 Department of Medicine, University of Alberta, Edmonton, Alberta T6G 2G3, Canada; 22 Liver Unit, Division of Gastroenterology and Hepatology, University of Calgary, Calgary, Alberta T6G 2X8, Canada; 23 Department of Medicine, Mount Sinai Hospital, Lunenfeld-Tanenbaum Research Institute, Toronto, Ontario M5G 1X5, Canada; 24 Liver Unit, Pomeranian Medical School, 71-252, Szczecin Poland; 25 Department of Optometry and Ophthalmology, Wenzhou Medical University, Wenzhou, Zhejiang 325035, China; 26 Division of Liver Diseases, Mount Sinai School of Medicine, New York, New York 10029, USA; 27 Department of Gastroenterology, Virginia Commonwealth University, Richmond, Virginia 23298-0341, USA; 28 Division of Gastroenterology and Hepatology, Saint Louis University School of Medicine, St Louis, Missouri 63110-0250, USA; 29 Department of Medicine, Beth Israel Medical Center, Albert Einstein College of Medicine, New York, New York, 10461, USA; 30 Centre of Hepatology, Gastroenterology and Dietetics, Vilnius University, Vilnius 01513, Lithuania; 31 Universite de Montreal Hospital Centre, Saint-Luc Hospital, Montreal, Quebec H2X 3J4, Canada; 32 Center for Liver Diseases Division of Hepatology, University of Miami School of Medicine, Miami, Florida 33136, USA; 33 Feinstein Institute for Medical Research, North Shore LIJ Health System, Manhasset, NY 11030, USA; 34 Gastroenterology & Hepatology Unit, Di.Bi.M.I.S., University of Palermo, Piazza delle Cliniche 2, Palermo 90127, Italy; 35 Eleonora Lorillard Spencer-Cenci Foundation, 00100, Rome, Italy; 36 Dipartimento di Scienze Mediche e Chirurgiche, Università di Bologna e Dipartimento dell'Apparato Digerente, Azienda Ospedaliero-Universitaria di Bologna, Policlinico Sant'Orsola Malpighi, via Massarenti 9, Bologna 40138, Italy; 37 Division of Gastroenterology, IRCCS Casa Sollievo della Sofferenza Hospital, viale Cappuccini 1, San Giovanni Rotondo 71013, Italy; 38 Department of Health Sciences, Università degli Studi di Milano, via di Rudinì, Milan 20142, Italy; 39 Liver Unit, Department of Health Sciences, San Paolo Hospital Medical School, University of Milan, via di Rudinì, Milan 20142, Italy; 40 Department of Gastroenterology, Università Politecnica delle Marche, Ospedali Riuniti University Hospital, via Tronto 10, Ancona 60126, Italy; 41 Center for Autoimmune Liver Diseases, Liver Unit, Department of Medicine, Humanitas Clinical and Research Center, via Manzoni 56, Rozzano, Milan 20089, Italy; 42 Department of Medico-Surgical Sciences and Biotechnologies, Polo Pontino, Sapienza University of Rome, Viale Università 37, 00185 Rome, Italy; 43 Hepatology Unit, Azienda Ospedaliera Universitaria Pisana, via Roma 67, Pisa 56126, Italy; 44 Gastroenterology and Liver Unit, Medical Department, Desio Hospital, via Mazzini, 5, Desio (MB) 20033, Italy; 45 Department of Internal Medicine, AO Provincia di Lecco, via dell'Eremo 9/11, Lecco 23900, Italy; 46 Division of Gastroenterology, Fondazione IRCCS Ca' Granda, Ospedale Maggiore Policlinico, Via Francesco Sforza 33, Milan 20122, Italy; 47 Liver Unit, Treviglio Hospital, P.le Ospedale 1, Treviglio (BG) 24047, Italy; 48 Clinica Patologie del Fegato, University of Trieste, & Fondazione Italiana Fegato (FIF), Cattinara Hospital, Trieste 34149, Italy; 49 Unit of Infectious Diseases and Hepatology, Azienda Ospedaliero-Universitaria di Parma, via Gramsci 14, Parma 43126, Italy; 50 Department of Molecular Medicine, University of Padova School of Medicine, Padova, Via Gabelli, 63, Padova 35121, Italy; 51 Department of Surgery, Oncology and Gastroenterology, University of Padova, via Giustiniani 2, Padua 35128, Italy; 52 Gastroenterology Unit, University of Florence, viale Pieraccini 6, Florence 50139, Italy; 53 Department of Experimental and Clinical Medicine, University of Florence, Largo Brambilla 3, Florence 50134, Italy; 54 SC Gastroenterologia Endoscopia Digestiva, Santa Croce Carle Hospital, via Coppino 26, Cuneo 12100, Italy; 55 Division of Gastroenterology, Sacro Cuore Don Calabria Hospital, Negrar, Verona 37024, Italy; 56 Division of Internal Medicine, Magenta Hospital, Via Al Donatore di Sangue 50, Magenta 20013, Italy; 57 Department of Clinical Medicine and Surgery, Gastroenterology Unit, University of Naples Federico II, 80138, Italy; 58 Department of Biomedical Sciences and Human Oncology, University Medical School, Bari, Piazza Giulio Cesare 11, Bari 70124, Italy; 59 Department of Internal Medicine, University of Genoa, viale Benedetto XV 6, Genoa 16145, Italy; 60 Division of Gastroenterology & Hepatology, Center for Predictive Medicine, Gradenigo Hospital, C.so Regina Margherita 10, Turin 10153, Italy; 61 Division of Gastroenterology, Valduce Hospital, via Dante 11, Como 22100, Italy; 62 Liver Center, Yale University School of Medicine, New Haven, Connecticut 06511, USA; 63 Section of Gastroenterology, Department of Translational Medicine and Surgery, University of Milan-Bicocca, via Pergolesi 33, Monza 20900, Italy; 64 Department of Internal Medicine, Liver Transplant Unit, University of Udine, P.zale S.M. Misericordia 1, Udine 33100, Italy; 65 Division of Hepatology, Niguarda Ca' Granda Hospital, Piazza Ospedale Maggiore 3, Milan 20162, Italy; 66 Abertawe Bro Morgannwg University Health Board, Morriston Hospital, Heol Maes Eglwys, Morriston SA6 6NL, Swansea UK; 67 Abertawe Bro Morgannwg University Health Board, Singleton Hospital, Sketty Lane, Sketty, Swansea SA2 8QA, UK; 68 Abertawe Bro Morgannwg University Health Board, Neath Port Talbot Hospital, Baglan Way, Port Talbot SA12 7BX, UK; 69 Abertawe Bro Morgannwg University Health Board, Princess of Wales Hospital, Coity Road, Bridgend CF31 1RQ, UK; 70 Aintree University Hospitals NHS Foundation Trust, Aintree University Hospital, Longmoor Lane, Liverpool L9 7AL UK; 71 Airedale NHS Foundation Trust, Airedale General Hospital, Skipton Road, Steeton, Keighley BD20 6TD UK; 72 Aneurin Bevan University Health Board, Nevill Hall Hospital, Brecon Road, Abergavenny NP7 7EG, UK; 73 Aneurin Bevan University Health Board, Royal Gwent Hospital, Cardiff Road, Newport NP20 2UB, UK; 74 Aneurin Bevan University Health Board, Ysbyty Ystrad Fawr, Ystrad Fawr Way, Ystrad Mynach, Hengoed CF82 7EP UK; 75 Ashford & St Peter's Hospitals NHS Foundation Trust, Ashford Hospital, London Road, Ashford TW15 3AA UK; 76 Ashford & St Peter's Hospitals NHS Foundation Trust, St Peter's Hospital, Guildford Road, Chertsey KT16 0PZ UK; 77 Barking, Havering and Redbridge University Hospitals NHS Trust, King George Hospital, Barley Lane, Ilford IG3 8YB UK; 78 Barking, Havering and Redbridge University Hospitals NHS Trust, Queen's Hospital, Rom Valley Way, Romford RM7 0AG UK, UK; 79 Barnsley Hospital NHS Foundation Trust, Barnsley Hospital, Gawber Road, Barnsley S75 2EP, UK; 80 Barts Health NHS Trust, The Royal London Hospital, Whitechapel Road, Whitechapel, London E1 1BB, UK; 81 Barts Health NHS Trust, Whipps Cross University Hospital, Whipps Cross Road, Leytonstone, London E11 1NR, UK; 82 Barts Health NHS Trust, Newham University Hospital, Glen Road, Plaistow, London E13 8SL, UK; 83 Basildon and Thurrock University Hospitals NHS Foundation Trust, Basildon University Hospital, Nethermayne, Basildon SS16 5NL, UK; 84 Bedford Hospital NHS Trust, Bedford Hospital, Kempston Road, Bedford MK42 9DJ, UK; 85 Belfast Health and Social Care Trust, Royal Victoria Hospital, 274 Grosvenor Road, Belfast BT12 6BA, UK; 86 Betsi Cadwaladr University Health Board, Glan Clwyd Hospital, Rhyl LL18 5UJ, UK; 87 Betsi Cadwaladr University Health Board, Ysbyty Gwynedd, Penrhosgarnedd, Bangor LL57 2PW, UK; 88 Betsi Cadwaladr University Health Board, Llandudno General Hospital, Hospital Road, Llandudno LL30 1LB, UK; 89 Betsi Cadwaladr University Health Board, Wrexham Maelor Hospital, Croesnewydd Road, Wrexham LL13 7TD, UK; 90 Blackpool Teaching Hospitals NHS Foundation Trusts, Blackpool Victoria Hospital, Whinney Heys Road, Blackpool FY3 8NR, UK; 91 Bolton NHS Foundation Trust, Royal Bolton Hospital, Minerva Road, Farnworth, Bolton BL4 0JR, UK; 92 Bradford Teaching Hospitals NHS Foundation Trust, Bradford Royal Infirmary, Duckworth Lane, Bradford BD9 6RJ, UK; 93 Brighton and Sussex University Hospitals NHS Trust, Princess Royal Hospital, Lewes Road, Haywards, Heath RH16 4EX, UK; 94 Brighton and Sussex University Hospitals NHS Trust, Royal Sussex County Hospital, Eastern Road, Brighton BN2 5BE, UK; 95 Buckinghamshire Healthcare NHS Trust, Amersham Hospital, Whielden Street, Amersham HP7 0JD, UK; 96 Buckinghamshire Healthcare NHS Trust, Stoke Mandeville Hospital, Mandeville Road, Aylesbury HP21 8AL, UK; 97 Buckinghamshire Healthcare NHS Trust, Wycombe Hospital, Queen Alexandra Road, High Wycombe HP11 2TT, UK; 98 Burton Hospitals NHS Foundation Trust, Queen's Hospital, Belvedere Road, Burton upon Trent DE13 0RB, UK; 99 Calderdale And Huddersfield NHS Foundation Trust, Calderdale Royal Hospital, Salterhebble, Halifax HX3 0PW, UK; 100 Calderdale And Huddersfield NHS Foundation Trust, Huddersfield Royal Infirmary, Acre Street, Lindley, Huddersfield HD3 3EA, UK; 101 Cambridge University Hospitals NHS Foundation Trust, Addenbrooke's Hospital, Cambridge Biomedical Campus, Hills Road, Cambridge CB2 0QQ, UK; 102 Cardiff and Vale University Health Board, University Hospital Llandough, Penlan Road, Llandough, Penarth CF64 2XX, UK; 103 Cardiff and Vale University Health Board, University Hospital of Wales, Heath Park, Cardiff CF14 4XW, UK; 104 Central Manchester University Hospitals NHS Foundation Trust, Manchester Royal Infirmary, Oxford Road, Manchester M13 9WL, UK; 105 Chelsea and Westminster Hospital NHS Foundation Trust, Chelsea and Westminster Hospital, 369 Fulham Road, London SW10 9NH, UK; 106 Chesterfield Royal Hospital NHS Foundation Trust, Chesterfield Royal Hospital, Calow, Chesterfield S44 5BL, UK; 107 City Hospitals Sunderland NHS Foundation Trust, Sunderland Royal Hospital, Kayll Road, Sunderland SR4 7TP, UK; 108 Colchester Hospital University NHS Foundation Trust, Colchester General Hospital, Turner Road, Colchester CO4 5JL, UK; 109 Countess of Chester Hospital NHS Foundation Trust, Countess of Chester Hospital, Countess of Chester Health Park, Liverpool Road, Chester CH2 1UL, UK; 110 County Durham and Darlington NHS Foundation Trust, Darlington Memorial Hospital, Hollyhurst Road, Darlington DL3 6HX, UK; 111 County Durham and Darlington NHS Foundation Trust, University Hospital of North Durham, North Road, Durham DH1 5TW, UK; 112 Croydon Health Services NHS Trust, Croydon University Hospital, 530 London Road, Croydon CR7 7YE, UK; 113 Croydon Health Services NHS Trust, Purley War Memorial Hospital, 856 Brighton Road, Purley CR8 2YL, UK; 114 Cwm Taf University Health Board, Prince Charles Hospital, Gurnos, Merthyr Tydfil CF47 9DT, UK; 115 Cwm Taf University Health Board, Royal Glamorgan Hospital, Ynysmaerdy, Llantrisant, Pontyclun CF72 8XR, UK; 116 Cwm Taf University Health Board, Ysbyty Cwm Cynon, New Road, Mountain Ash, Rhondda Cynon Taff CF45 4BZ, UK; 117 Dartford And Gravesham NHS Trust, Darent Valley Hospital, Darenth Wood Road, Dartford DA2 8DA, UK; 118 Oxleas NHS Foundation Trust, Queen Mary's Hospital Sidcup, Frognal Avenue, Sidcup DA14 6LT UK; 119 Derby Hospitals NHS Foundation Trust, Royal Derby Hospital, Uttoxeter Road, Derby DE22 3NE, UK; 120 Doncaster and Bassetlaw Hospitals NHS Foundation Trust, Bassetlaw Hospital, Blyth Road, Worksop S81 0BD, UK; 121 Doncaster and Bassetlaw Hospitals NHS Foundation Trust, Doncaster Royal Infirmary, Armthorpe Road, Doncaster DN2 5LT, UK; 122 Dorset County Hospitals NHS Foundation Trust, Dorset County Hospital, Williams Avenue, Dorchester DT1 2JY, UK; 123 Dudley Group of Hospitals NHS Trust, Russells Hall Hospital, Pensnett Road, Dudley DY1 2HQ, UK; 124 East and North Hertfordshire NHS Trust, Lister Hospital, Coreys Mill Lane, Stevenage SG1 4AB, UK; 125 East and North Hertfordshire NHS Trust, Queen Elizabeth II Hospital, Howlands, Welwyn Garden City AL7 4HQ, UK; 126 East Cheshire NHS Trust, Macclesfield District General Hospital, Victoria Road, Macclesfield SK10 3BL, UK; 127 East Kent Hospitals University NHS Foundation Trust, Buckland Hospital, Coombe Valley Road, Dover CT17 0HD, UK; 128 East Kent Hospitals University NHS Foundation Trust, Kent and Canterbury Hospital, Ethelbert Road, Canterbury CT1 3NG, UK; 129 East Kent Hospitals University NHS Foundation Trust, Queen Elizabeth The Queen Mother Hospital, St Peters Road, Margate CT9 4AN, UK; 130 East Kent Hospitals University NHS Foundation Trust, William Harvey Hospital Ashford, Kennington Road, Willesborough, Ashford TN24 0LZ, UK; 131 East Lancashire Hospitals NHS Trust, Burnley General Hospital, Casterton Avenue, Burnley BB10 2PQ, UK; 132 East Lancashire Hospitals NHS Trust, Royal Blackburn Hospital, Haslingden Road, Blackburn BB2 3HH, UK; 133 East Sussex Healthcare NHS Trust, Conquest Hospital, The Ridge, St Leonards-on-Sea TN37 7RD, UK; 134 East Sussex Healthcare NHS Trust, Eastbourne District General Hospital, Kings Drive, Eastbourne BN21 2UD; 135 Epsom and St Helier University Hospitals NHS Trust, Epsom General Hospital, Epsom Hospital, Dorking Road, Epsom KT18 7EG, UK; 136 Frimley Health NHS Foundation Trust, Heatherwood Hospital, London Road, Ascot SL5 8AA, UK; 137 Frimley Health NHS Foundation Trust, Wexham Park Hospital, Wexham, Slough SL2 4HL, UK; 138 Frimley Health NHS Foundation Trust, Frimley Park Hospital, Portsmouth Road, Frimley GU16 7UJ, UK; 139 Gateshead Health NHS Foundation Trust, Queen Elizabeth Hospital, Sheriff Hill, Gateshead NE9 6SX, UK; 140 George Eliot Hospital NHS Trust, George Eliot Hospital, Eliot Way, Nuneaton CV10 7DJ, UK; 141 Gloucestershire Hospitals NHS Foundation Trust, Cheltenham General Hospital, Sandford Road, Cheltenham GL53 7AN, UK; 142 Gloucestershire Hospitals NHS Foundation Trust, Gloucestershire Royal Hospital, Great Western Road, Gloucester GL1 3NN, UK; 143 Guy's and St Thomas' NHS Foundation Trust, Guy's Hospital, Great Maze Pond, London SE1 9RT, UK; 144 Guy's and St Thomas' NHS Foundation Trust, St Thomas' Hospital, Westminster Bridge Road, London SE1 7EH, UK; 145 Hampshire Hospitals NHS Foundation Trust, Royal Hampshire County Hospital, Romsey Road, Winchester SO22 5DG, UK; 146 Hampshire Hospitals NHS Foundation Trust, Basingstoke and North Hampshire Hospital, Aldermaston Road, Basingstoke RG24 9NA, UK; 147 Harrogate and District NHS Foundation Trust, Harrogate District Hospital, Lancaster Park Road, Harrogate HG2 7SX, UK; 148 Heart of England NHS Foundation Trust, Good Hope Hospital, Rectory Road, Sutton Coldfield, Birmingham B75 7RR UK; 149 Heart of England NHS Foundation Trust, Heartlands Hospital, Bordesley Green East, Birmingham B9 5SS, UK; 150 Heart of England NHS Foundation Trust, Solihull Hospital, Lode Lane, Solihull B91 2JL, UK; 151 Hillingdon Hospitals NHS Foundation Trust, Hillingdon Hospital, Pield Heath Road, Uxbridge UB8 3NN, UK; 152 Hinchingbrooke Health Care NHS Trust, Hinchingbrooke Hospital, Hinchingbrooke Park, Huntingdon PE29 6NT, UK; 153 Homerton University Hospital NHS Foundation Trust, Homerton University Hospital, Homerton Row, London E9 6SR, UK; 154 Hull And East Yorkshire Hospitals NHS Trust, Castle Hill Hospital, Castle Road, Cottingham HU16 5JQ, UK; 155 Hull And East Yorkshire Hospitals NHS Trust, Hull Royal Infirmary, Anlaby Road, Hull HU3 2JZ, UK; 156 Hywel Dda University Health Board, Withybush General Hospital, Fishguard Road, Haverfordwest SA61 2PZ, UK; 157 Hywel Dda University Health Board, Prince Philip Hospital, Bryngwyn Mawr, Dafen, Llanelli SA14 8QF, UK; 158 Hywel Dda University Health Board, Glangwili General Hospital, Dolgwilli Road, Carmarthen SA31 2AF, UK; 159 Hywel Dda University Health Board, Bronglais Hospital, Caradog Road, Aberystwyth SY23 1ER, UK; 160 Imperial College Healthcare NHS Trust, Charing Cross Hospital, Fulham Palace Road, London W6 8RF, UK; 161 Imperial College Healthcare NHS Trust, Hammersmith Hospital, Du Cane Road, London W12 0HS, UK; 162 Imperial College Healthcare NHS Trust, St Mary's Hospital, Praed Street, London W2 1NY, UK; 163 Ipswich Hospital NHS Trust, Ipswich Hospital, Heath Road, Ipswich IP4 5PD, UK; 164 Isle of Wight NHS Trust, St Mary's Hospital, Parkhurst Road, Newport PO30 5TG, UK; 165 James Paget University Hospitals NHS Foundation Trust, James Paget Hospital, Lowestoft Road, Gorleston, Great Yarmouth NR31 6LA, UK; 166 Kettering General Hospital NHS Foundation Trust, Kettering General Hospital, Rothwell Road, Kettering NN16 8UZ, UK; 167 Kings College Hospital NHS Foundation Trust, King's College Hospital, Denmark Hill, London SE5 9RS, UK; 168 King's College Hospital NHS Foundation Trust, Beckenham Beacon, 395 Croydon Road, Beckenham BR3 3QL, UK; 169 King's College Hospital NHS Foundation Trust, Princess Royal University Hospital, Farnborough Common, Orpington BR6 8ND, UK; 170 Kingston Hospital NHS Foundation Trust, Kingston Hospital, Galsworthy Road, Kingston upon Thames KT2 7QB, UK; 171 Lancashire Teaching Hospitals NHS Foundation Trust, Chorley and South Ribble Hospital, Preston Road, Chorley PR7 1PP, UK; 172 Lancashire Teaching Hospitals NHS Foundation Trust, Royal Preston Hospital, Sharoe Green Lane North, Preston PR2 9HT, UK; 173 Leeds Teaching Hospitals NHS Trust, Leeds General Infirmary, Great George Street, Leeds LS1 3EX, UK; 174 Leeds Teaching Hospitals NHS Trust, St James's University Hospital, Beckett Street, Leeds LS9 7TF, UK; 175 Lewisham and Greenwich NHS Trust, The Queen Elizabeth, Woolwich, Stadium Road, Greenwich SE18 4QH, UK; 176 Lewisham and Greenwich NHS Trust, Lewisham Hospital, High Street, Lewisham SE13 6LH, UK; 177 London North West Healthcare NHS Trust, Central Middlesex Hospital, Acton Lane, Park Royal, London NW10 7NS, UK; 178 London North West Healthcare NHS Trust, Northwick Park and St Mark's Hospitals, Watford Road, Harrow HA1 3UJ, UK; 179 Luton and Dunstable University Hospital NHS Foundation Trust, Luton and Dunstable University Hospital, Lewsey Road, Luton LU4 0DZ, UK; 180 Maidstone and Tunbridge Wells NHS Trust, Maidstone Hospital, Hermitage Lane, Maidstone ME16 9QQ, UK; 181 Maidstone and Tunbridge Wells NHS Trust, Tunbridge Wells Hospital, Tonbridge Road, Pembury, Tunbridge Wells TN2 4QJ, UK; 182 Medway NHS Foundation Trust, Medway Maritime Hospital, Windmill Road, Gillingham ME7 5NY UK; 183 Mid Cheshire Hospitals NHS Foundation Trust, Leighton Hospital, Middlewich Road, CW1 4QJ, UK; 184 Mid Essex Hospital Services NHS Trust, Broomfield Hospital, Court Road, Chelmsford CM1 7ET, UK; 185 Mid Essex Hospital Services NHS Trust, St Peters Hospital, Spital Road, Maldon CM9 6EG, UK; 186 Mid Yorkshire Hospitals NHS Trust, Dewsbury and District Hospital, Halifax Road, Dewsbury WF13 4HS, UK; 187 Milton Keynes Hospital NHS Foundation Trust, Milton Keynes Hospital, Standing Way, Milton Keynes MK6 5LD, UK; 188 Newcastle upon Tyne Hospitals NHS Foundation Trust, Freeman Hospital, Freeman Road, High Heaton, Newcastle upon Tyne NE7 7DN, UK; 189 NHS Ayrshire & Arran, University Hospital Crosshouse, Kilmarnock Road, Kilmarnock KA2 0BE, UK; 190 NHS Borders, Borders General Hospital, Melrose TD6 9BS, UK; 191 NHS Dumfries & Galloway, Dumfries and Galloway Royal Infirmary, Bankend Road, Dumfries DG1 4AP, UK; 192 NHS Fife, Queen Margaret Hospital, Whitefield Road, Dunfermline KY12 0SU, UK; 193 NHS Fife, Victoria Hospital, Hayfield Road, Kirkcaldy KY2 5AH, UK; 194 NHS Forth Valley, Forth Valley Royal Hospital, Stirling Road, Larbert FK5 4WR, UK; 195 NHS Forth Valley, Stirling Community Hospital, Livilands, Stirling FK8 2AU, UK; 196 NHS Grampian, Aberdeen Royal Infirmary, Foresterhill, Aberdeen AB25 2ZN, UK; 197 NHS Grampian, Dr Gray's Hospital, Elgin IV30 1SN, UK; 198 NHS Grampian, Woolmanhill Hospital, Skene Street, Aberdeen AB25 1LD, UK; 199 NHS Greater Glasgow and Clyde, Gartnavel General Hospital, 1053 Great Western Road, Glasgow G12 0YN, UK; 200 NHS Greater Glasgow and Clyde, Glasgow Royal Infirmary, 84 Castle Street, Glasgow G4 0SF, UK; 201 NHS Greater Glasgow and Clyde, Inverclyde Royal Hospital, Larkfield Road, Greenock PA16 0XN, UK; 202 NHS Greater Glasgow and Clyde, Royal Alexandra Hospital, Corsebar Road, Paisley PA2 9PN, UK; 203 NHS Greater Glasgow and Clyde, Southern General Hospital, 1345 Govan Road, Glasgow G51 4TF, UK; 204 NHS Greater Glasgow and Clyde, Victoria Infirmary, Langside Road, Glasgow G42 9TY, UK; 205 NHS Highland, Caithness General Hospital, Bankhead Road, Wick KW1 5NS, UK; 206 NHS Highland, Raigmore Hospital, Old Perth Road, Inverness IV2 3UJ, UK; 207 NHS Lanarkshire, Hairmyres Hospital, Eaglesham Road, East Kilbride G75 8RG, UK; 208 NHS Lanarkshire, Monklands Hospital, Monkscourt Avenue, Airdrie ML6 0JS, UK; 209 NHS Lanarkshire, Wishaw General Hospital, 50 Netherton Street, Wishaw ML2 0DP, UK; 210 NHS Lothian, Royal Infirmary of Edinburgh, 51 Little France Crescent, Old Dalkeith Road, Edinburgh EH16 4SA, UK; 211 NHS Lothian, St John's Hospital, Howden Road West, Howden, Livingston EH54 6PP, UK; 212 NHS Lothian, Western General Hospital, Crewe Road South, Edinburgh EH4 2XU, UK; 213 NHS Tayside, Perth Royal Infirmary, Taymount Terrace, Perth PH1 1NX, UK; 214 NHS Tayside, Ninewells Hospital, Dundee DD1 9SY, UK; 215 Norfok and Norwich University Hospitals NHS Foundation Trust, Norfolk and Norwich University Hospital, Colney Lane, Norwich NR4 7UY, UK; 216 North Bristol NHS Trust, Frenchay Hospital, Frenchay Park Road, Bristol BS16 1LE, UK; 217 North Cumbria University Hospitals NHS Foundation Trust, Cumberland Infirmary, Newtown Road, Carlisle CA2 7HY, UK; 218 North Cumbria University Hospitals NHS Foundation Trust, West Cumberland Hospital, Hensingham, Whitehaven CA28 8JG, UK; 219 North Tees and Hartlepool NHS Foundation Trust, University Hospital of Hartlepool, Holdforth Road, Hartlepool TS24 9AH, UK; 220 North Tees and Hartlepool NHS Foundation Trust, University Hospital of North Tees, Hardwick, Stockton on Tees TS19 8PE, UK; 221 Northampton General Hospital NHS Trust, Northampton General Hospital, Cliftonville, Northampton NN1 5BD, UK; 222 Northern Devon Healthcare NHS Trust, North Devon District Hospital, Raleigh Park, Barnstaple EX31 4JB, UK; 223 Northern Health and Social Care Trust, Whiteabbey Hospital, Doagh Road, Newtownabbey BT37 9RH, UK; 224 Northern Lincolnshire and Goole NHS Foundation Trust, Diana, Princess of Wales Hospital, Scartho Road, Grimsby DN33 2BA, UK; 225 Northern Lincolnshire and Goole NHS Foundation Trust, Goole and District Hospital, Woodland Avenue, Goole DN14 6RX, UK; 226 Northern Lincolnshire and Goole NHS Foundation Trust, Scunthorpe General Hospital, Cliff Gardens, Scunthorpe DN15 7BH, UK; 227 Northumbria Healthcare NHS Foundation Trust, Hexham General Hospital, Corbridge Road, Hexham NE46 1QJ, UK; 228 Northumbria Healthcare NHS Foundation Trust, North Tyneside Hospital, Rake Lane, North Shields NE29 8NH, UK; 229 Nottingham University Hospitals NHS Trust, Nottingham City Hospital, Hucknall Road, Nottingham NG5 1PB, UK; 230 Nottingham University Hospitals NHS Trust, Queen's Medical Centre, Derby Road, Nottingham NG7 2UH UK; 231 Oxford University Hospitals NHS Trust, John Radcliffe Hospital, Headley Way, Headington, Oxford OX3 9DU, UK; 232 Pennine Acute Hospitals NHS Trust, Fairfield General Hospital, Rochdale Old Road, Bury BL9 7TD, UK; 233 Pennine Acute Hospitals NHS Trust, North Manchester General Hospital, Delaunays Road, Crumpsall M8 5RB, UK; 234 Pennine Acute Hospitals NHS Trust, Rochdale Infirmary, Whitehall Street, Rochdale OL12 0NB, UK; 235 Pennine Acute Hospitals NHS Trust, The Royal Oldham Hospital, Rochdale Road, Oldham OL1 2JH, UK; 236 Peterborough and Stamford Hospitals NHS Foundation Trust, Peterborough City Hospital, Edith Cavell Campus, Bretton Gate, Peterborough PE3 9GZ, UK; 237 Peterborough and Stamford Hospitals NHS Foundation Trust, Stamford & Rutland Hospital, Ryhall Road, Stamford PE9 1UA, UK; 238 Plymouth Hospitals NHS Trust, Derriford Hospital, Derriford Road, Plymouth PL6 8DH, UK; 239 Poole Hospital NHS Foundation Trust, Poole Hospital, Longfleet Road, Poole BH15 2JB, UK; 240 Portsmouth Hospitals NHS Trust, Queen Alexandra Hospital, Cosham, Portsmouth PO6 3LY, UK; 241 Princess Alexandra Hospital NHS Trust, St Margaret's Hospital, The Plain, Epping CM16 6TN, UK; 242 Princess Alexandra Hospital NHS Trust, The Princess Alexandra Hospital, Hamstel Road, Harlow CM20 1QX, UK; 243 Queen Elizabeth Hospital King's Lynn NHS Foundation Trust, The Queen Elizabeth Hospital King's Lynn, Gayton Road, King's Lynn PE30 4ET, UK; 244 Rotherham NHS Foundation Trust, Rotherham Hospital, Moorgate Road, Rotherham S60 2UD, UK; 245 Royal Berkshire NHS Foundation Trust, Royal Berkshire Hospital, Craven Road, Reading RG1 5AN, UK; 246 Royal Bournemouth and Christchurch Hospitals NHS Foundation Trust, Royal Bournemouth Hospital, Castle Lane East, Bournemouth BH7 7DW, UK; 247 Royal Cornwall Hospitals NHS Trust, Royal Cornwall Hospital, Treliske, Truro TR1 3LJ, UK; 248 Royal Devon and Exeter NHS Foundation Trust, Royal Devon and Exeter Hospital, Barrack Road, Exeter EX2 5DW, UK; 249 Royal Free London NHS Foundation Trust, The Royal Free Hospital, Pond Street, London NW3 2QG, UK; 250 Royal Free London NHS Foundation Trust, Barnet Hospital, Wellhouse Lane, Barnet EN5 3DJ, UK; 251 Royal Free London NHS Foundation Trust, Chase Farm Hospital, The Ridgeway, Enfield EN2 8JL, UK; 252 Royal Liverpool and Broadgreen University Hospitals NHS Trust, Royal Liverpool University Hospital, Prescot Street, Liverpool L7 8XP, UK; 253 Royal United Hospitals Bath NHS Foundation Trust, Royal United Bath Hospital, Combe Park, Bath BA1 3NG, UK; 254 Royal Wolverhampton Hospitals NHS Trust, New Cross Hospital, Wolverhampton Road, Wolverhampton WV10 0QP, UK; 255 Royal Wolverhampton Hospitals NHS Trust, Cannock Chase Hospital, Brunswick Road, Cannock WS11 5XY, UK; 256 University Hospitals of North Midlands NHS Trust, County Hospital, Weston Road, Stafford ST16 3SA, UK; 257 Salisbury NHS Foundation Trust, Salisbury District Hospital, Salisbury SP2 8BJ, UK; 258 Sandwell and West Birmingham Hospitals NHS Trust, Sandwell General Hospital, Lyndon, West Bromwich B71 4HJ, UK; 259 Sheffield Teaching Hospitals NHS Foundation Trust, Northern General Hospital, Herries Road, Sheffield S5 7AU, UK; 260 Sheffield Teaching Hospitals NHS Foundation Trust, Royal Hallamshire Hospital, Glossop Road, Sheffield S10 2JF, UK; 261 Sherwood Forest Hospitals NHS Foundation Trust, King's Mill Hospital, Mansfield Road, Sutton in Ashfield NG17 4JL, UK; 262 Sherwood Forest Hospitals NHS Foundation Trust, Newark Hospital, Boundary Road, Newark NG24 4DE, UK; 263 Shrewsbury and Telford Hospital NHS Trust, Princess Royal Hospital, Apley Castle, Telford TF1 6TF, UK; 264 Shrewsbury and Telford Hospital NHS Trust, Royal Shrewsbury Hospital, Mytton Oak Road, Shrewsbury SY3 8XQ, UK; 265 South Devon Healthcare NHS Foundation Trust, Torbay Hospital, Lowes Bridge, Torquay TQ2 7AA, UK; 266 South Eastern Health and Social Care Trust, Lagan Valley Hospital, 39 Hillsborough Road, Lisburn BT28 1JP, UK; 267 South Eastern Health and Social Care Trust, Ulster Hospital, Upper Newtownards Road, Dundonald, Belfast BT16 1RH, UK; 268 South Tees Hospitals NHS Foundation Trust, The James Cook University Hospital, Marton Road, Middlesbrough TS4 3BW, UK; 269 South Tees Hospitals NHS Foundation Trust, Friarage Hospital, Northallerton DL6 1JG, UK; 270 South Tyneside NHS Foundation Trust, South Tyneside District Hospital, Harton Lane, South Shields NE34 0PL, UK; 271 South Warwickshire NHS Foundation Trust, Warwick Hospital, Lakin Road, Warwick CV34 5BW, UK; 272 Southend University Hospital NHS Foundation Trust, Southend Hospital, Prittlewell Chase, Westcliff-on-Sea SS0 0RY, UK; 273 Southport & Ormskirk Hospital NHS Trust, Ormskirk District General Hospital, Wigan Road, Ormskirk L39 2AZ, UK; 274 Southport & Ormskirk Hospital NHS Trust, Southport and Formby District General Hospital, Town Lane, Kew, Southport PR8 6PN, UK; 275 St George's University Hospitals NHS Foundation Trust, St George's Hospital, Blackshaw Road, Tooting, London SW17 0QT, UK; 276 St Helens and Knowsley Teaching Hospitals NHS Trust, St Helens Hospital, Marshalls Cross Road, St Helens WA9 3DA, UK; 277 St Helens and Knowsley Teaching Hospitals NHS Trust, Whiston Hospital, Warrington Road, Prescot L35 5DR, UK; 278 Stockport NHS Foundation Trust, Stepping Hill Hospital, Poplar Grove, Hazel Grove, Stockport SK2 7JE, UK; 279 Surrey and Sussex Healthcare NHS Trust, East Surrey Hospital, Canada Avenue, Redhill RH1 5RH, UK; 280 Tameside Hospital NHS Foundation Trust, Tameside General Hospital, Fountain Street, Ashton-under-Lyne OL6 9RW, UK; 281 United Lincolnshire Hospitals NHS Trust, Lincoln County Hospital, Greetwell Road, Lincoln LN2 5QY, UK; 282 United Lincolnshire Hospitals NHS Trust, Grantham and District Hospital, 101 Manthorpe Road, Grantham NG31 8DG, UK; 283 United Lincolnshire Hospitals NHS Trust, Pilgrim Hospital Boston, Sibsey Road, Boston PE21 9QS, UK; 284 University College London Hospitals NHS Foundation Trust, University College Hospital, 235 Euston Road, London NW1 2BU, UK; 285 University Hospital of South Manchester NHS Foundation Trust, Wythenshawe Hospital, Southmoor Road, Wythenshawe, Manchester M23 9LT, UK; 286 University Hospital Southampton NHS Foundation Trust, Southampton General Hospital, Tremona Road, Southampton SO16 6YD, UK; 287 University Hospitals Birmingham NHS Foundation Trust, Queen Elizabeth Hospital, Mindelsohn Way, Edgbaston, Birmingham B15 2GW, UK; 288 University Hospitals Bristol NHS Foundation Trust, Bristol Royal Infirmary, Upper Maudlin Street, Bristol BS2 8HW, UK; 289 University Hospitals Coventry and Warwickshire NHS Trust, University Hospital, Clifford Bridge Road, Coventry CV2 2DX, UK; 290 University Hospitals of Leicester NHS Trust, Glenfield Hospital, Groby Road, Leicester LE3 9QP, UK; 291 University Hospitals of Leicester NHS Trust, Leicester General Hospital, Gwendolen Road, Leicester LE5 4PW, UK; 292 University Hospitals of Leicester NHS Trust, Leicester Royal Infirmary, Infirmary Square, Leicester LE1 5WW, UK; 293 University Hospitals of Morecambe Bay NHS Foundation Trust, Royal Lancaster Infirmary, Ashton Road, Lancaster LA1 4RP, UK; 294 University Hospitals of North Midlands NHS Trust, Royal Stoke University Hospital, Newcastle Road, Stoke-on-Trent ST4 6QG, UK; 295 Walsall Healthcare NHS Trust, Walsall Manor Hospital, Moat Road, Walsall WS2 9PS, UK; 296 Warrington and Halton Hospitals NHS Foundation Trust, Warrington Hospital, Lovely Lane, Warrington WA5 1QG, UK; 297 West Hertfordshire Hospitals NHS Trust, Hemel Hempstead General Hospital, Hillfield Road, Hemel Hempstead HP2 4AD, UK; 298 West Hertfordshire Hospitals NHS Trust, St Albans City Hospital, Waverley Road, St Albans AL3 5PN, UK; 299 West Hertfordshire Hospitals NHS Trust, Watford General Hospital, Vicarage Road, Watford WD18 0HB, UK; 300 West Middlesex University NHS Trust, West Middlesex University Hospital, Twickenham Road, Isleworth TW7 6AF; 301 West Suffolk NHS Foundation Trust, Walnut Tree Hospital, Walnut Tree Lane, Sudbury CO10 1BE, UK; 302 West Suffolk NHS Foundation Trust, West Suffolk Hospital, Hardwick Lane, Bury St Edmunds IP33 2QZ, UK; 303 Western Sussex Hospitals NHS Foundation Trust, Worthing Hospital, Lyndhurst Road, Worthing BN11 2DH, UK; 304 Western Sussex Hospitals NHS Foundation Trust, St Richard's Hospital, Spitalfield Lane, Chichester PO19 6SE, UK; 305 Weston Area Health NHS Trust, Weston General Hospital, Grange Road, Uphill, Weston super Mare BS23 4TQ, UK; 306 Whittington Hospital NHS Trust, The Whittington Hospital, Magdala Avenue, London N19 5NF, UK; 307 Wirral University Teaching Hospital NHS Foundation Trust, Arrowe Park Hospital, Upton CH49 5PE, UK; 308 Wirral University Teaching Hospital NHS Foundation Trust, Victoria Central Hospital, Mill Lane, Wallasey CH44 5UF, UK; 309 Worcestershire Acute Hospitals NHS Trust, Alexandra Hospital, Woodrow Drive, Redditch B98 7UB, UK; 310 Worcestershire Acute Hospitals NHS Trust, Kidderminster Hospital and Treatment Centre, Bewdley Road, Kidderminster DY11 6RJ, UK; 311 Worcestershire Acute Hospitals NHS Trust, Worcestershire Royal Hospital, Charles Hastings Way, Worcester WR5 1DD, UK; 312 Wrightington, Wigan And Leigh NHS Trust, Royal Albert Edward Infirmary, Wigan Lane, Wigan WN1 2NN, UK; 313 Wye Valley NHS Trust, The County Hospital, Stonebow Road, Hereford HR1 2BN, UK; 314 Yeovil District Hospital NHS Foundation Trust, Yeovil District Hospital, Higher Kingston, Yeovil BA21 4AT, UK; 315 York Teaching Hospital NHS Foundation Trust, Bridlington Hospital, Bessingby Road, Bridlington YO16 4QP, UK; 316 York Teaching Hospital NHS Foundation Trust, Scarborough Hospital, Woodlands Drive, Scarborough YO12 6QL, UK; 317 York Teaching Hospital NHS Foundation Trust, The York Hospital, Wigginton Road, York YO31 8HE, UK; 318 Great Western Hospitals NHS Foundation Trust, Marlborough Road, Swindon, Wiltshire SN3 6BB, UK

## Abstract

Primary biliary cirrhosis (PBC) is a classical autoimmune liver disease for which effective immunomodulatory therapy is lacking. Here we perform meta-analyses of discovery data sets from genome-wide association studies of European subjects (*n*=2,764 cases and 10,475 controls) followed by validation genotyping in an independent cohort (*n*=3,716 cases and 4,261 controls). We discover and validate six previously unknown risk loci for PBC (*P*_combined_<5 × 10^−8^) and used pathway analysis to identify JAK-STAT/IL12/IL27 signalling and cytokine–cytokine pathways, for which relevant therapies exist.

## Abstract (web summary)

Primary biliary cirrhosis is an autoimmune liver disease with poor therapeutic options. Here Cordell *et al*. a perform meta-analysis of European genome-wide association studies identifying six novel risk loci and a number of potential therapeutic pathways.

## Graphical abstract

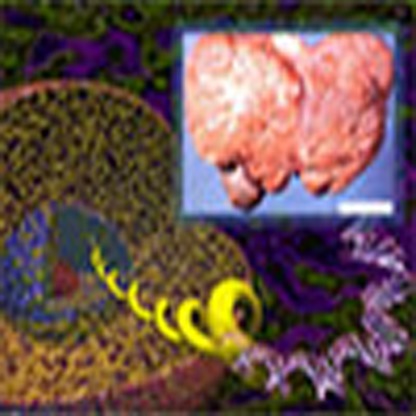

Primary biliary cirrhosis (PBC) is a rare cholestatic liver disease characterized by progressive autoimmune destruction of intrahepatic bile ducts, leading to cirrhosis and liver failure in a substantial proportion of cases^1^. To date, four genome-wide association studies (GWAS) and two Illumina immunochip studies of PBC have confirmed associations at the human leukocyte antigen (HLA) locus and identified 27 non-HLA risk loci^2^,^3^,^4^,^5^,^6^,^7^,^8^. Consistent with GWAS data for other autoimmune diseases, results of these studies implicate immune-related genes in disease pathogenesis, but in general fail to pinpoint the disease-causal variants within the identified risk loci. To identify risk alleles that may be relevant to disease biology and treatment and illuminate additional PBC risk loci, we undertook a genome-wide meta-analysis (GWMA) combining North American, Italian and UK PBC GWAS data sets^2^,^4^,^5^. Functional annotation of the risk loci and pathway analyses were then performed to identify the alleles and pathways most relevant to disease cause and treatment.

## Results

### Discovery of new PBC risk loci

Following quality control, the combined discovery data set for GWMA consisted of 1,143,634 genotyped or imputed single-nucleotide polymorphisms (SNPs) in 2,764 cases and 10,475 controls (Supplementary Table 1). After genomic control correction and exclusion of known PBC risk loci from the final set of results, the inflation factor was *λ*=1.043 (Supplementary Fig. 1). Meta-analysis of this data set identified 23 loci at genome-wide level of significance (*P*<5 × 10^−8^, calculated using logistic regression of individual discovery data sets in ProbABEL followed by genomic control correction of individual discovery data sets in R and fixed-effects meta-analysis in META, see Methods). Of these, 22 had been detected in previous studies and the 23rd corresponded to a most-likely spurious signal from a single imputed SNP on chromosome 13 (Supplementary Fig. 2, Supplementary Table 2). However, we found suggestive evidence of association (*P*<2 × 10^−5^ from fixed-effects meta-analysis in META) at 41 loci not previously known to be associated with PBC. The top-scoring SNPs (or close proxies in strong linkage disequilibrium with the top-scoring SNP) from these and nine other loci (including the likely spurious chromosome 13 signal) were taken forward for genotyping in an independent panel consisting of 3,716 PBC cases and 4,261 controls. In total, 120 SNPs at 50 independent loci were taken forward for validation, of which 114 were successfully genotyped (Supplementary Data 1).

In the validation analysis, we confirmed association with SNPs at six loci not previously known to be associated with PBC (*P*<4.4 × 10^−4^, equivalent to *P=*0.05 with a Bonferroni correction for 114 tests, calculated using logistic regression analysis of individual validation data sets in PLINK followed by meta-analysis in META, see Methods); meta-analysis of discovery and validation cohorts at these loci reached genome-wide levels of significance (*P*_combined_ <5 × 10^−8^ from fixed-effect meta-analysis in META) (Table 1, Supplementary Figs 3 and 4). Furthermore, SNPs at two additional loci achieved *P* values suggestive of association (*P*<1 × 10^−3^ from fixed-effect meta-analysis in META, equivalent to *P*=0.05 with a Bonferroni correction for testing at 50 independent loci; Table 1, Supplementary Fig. 5). Newly identified PBC risk loci overlap with those of other autoimmune disorders and harbour several immunologically relevant candidate genes, most notably chemokine ligand 20 (*CCL20*) and interleukin 12B (*IL12B*; Table 1).

### Discovery of candidate causal disease variants

In functional annotation of risk loci, we identified 199 candidate variants across 28 non-HLA risk loci with probabilistic identification of causal SNPs (PICS) probability >0.0275 (ref. 9). At each risk locus, the most-likely causal variant was the index variant, with median PICS probability of 0.224 and values up to 0.998 for rs2546890 at 5q33.3 (Supplementary Data 2). Looking at all candidate variants across all risk loci, the majority were intronic, upstream or downstream gene variants with no predicted functional consequence (99/199, 40%). However, a substantial proportion (59/199, 30%) were regulatory region variants, defined as SNPs located within regulatory features, including enhancers, promoters, transcription factor-binding sites and open chromatin regions (Supplementary Data 3). Notably, candidate variants at 18 (64%) of the 28 annotated risk loci included at least one regulatory region variant. In contrast, only 5 of 199 candidates were missense variants (2.5%) (Supplementary Table 3a). However, these included rs2297067 in *EXOC3L4* at 14q32.32 and rs2304256 in *TYK2* at 19p13.2, both predicted by SIFT and/or PolyPhen to be deleterious or potentially damaging^10^,^11^. Candidate variants included a single splice region variant, that is, rs17641524 at 1q31.3 that is predicted to affect splicing of *DENND1B* (Supplementary Table 3b).

We found that candidate variants at several risk loci are methylation quantitative trait loci (mQTLs), including mQTLs for *DENNDIB*, *PLCL2*, *IRF5* and *TNFRSF1A*, all genes that are implicated in risk for other autoimmune diseases (Supplementary Data 4). We also found that candidate variants at several risk loci are expression quantitative trait loci (eQTLs) in lymphoblastoid and other cell lineages, including eQTLs for *CCL20*, *IL12A*, *IRF5* and *TYK2* (Supplementary Data 5).

At many risk loci, functional annotation highlighted a single candidate gene (Supplementary Data 2). However, most risk loci contained multiple compelling candidate variants. This complexity is well illustrated by the composite of candidate variants at the *PLCL2* gene and *MANBA* gene loci, which include multiple eQTL and mQTL SNPs. Thus, despite the presence of many candidate variants with regulatory or epigenetic roles within PBC risk loci, more direct biological experimental approaches are required to pinpoint the disease-causal variants at these loci.

We also applied functional GWAS (FGWAS) and its associated annotation file^12^ to our full set of discovery GWMA results and thereby identified 75 annotations with enrichment (*P*<0.01 from FGWAS) of GWMA association signals (Supplementary Data 6). After a stepwise selection approach similar to that of Pickrell^12^, the best-fitting model included six annotations highlighting negative enrichment of repressed chromatin regions in a lymphoblastoid cell line, and positive enrichment of DNase-I-hypersensitive sites in a variety of cell types, in particular CD20+ and Th1 T cells (Supplementary Table 4).

### Identification of candidate targetable biological pathways

To identify biological pathways involved in development of PBC, we conducted pathway analysis using GCTA^13^ followed by i-GSEA4GWAS^14^. We identified several immunoregulatory pathways associated with PBC, in particular, IL-12 and other cytokines as well as T-cell signalling pathways. To account for bias that might result from the strong HLA association with PBC, we repeated this analysis with SNPs/genes in the HLA region excluded. Notably, IL-12, IL-27 and JAK-STAT signalling pathways were still associated with PBC, even after their HLA contribution had been removed (Table 2).

We identified molecules that targeted these pathways by overlaying the Drug Gene Interaction database^15^ and calculating a pathway specificity score and Jaccard index of each drug for each of the pathways that remained associated with PBC after the HLA contribution had been removed (Table 2, Supplementary Data 7). This combined analysis identified pathways and immunomodulatory agents that represent promising leads for further study in models of PBC.

## Discussion

The current study adds to our knowledge of the genetic architecture of PBC. Notably, our data identify *CCL20* as a candidate risk gene for PBC. Chemokine ligand 20 (CCL20) and its chemokine receptor CCR6 contribute to the formation and function of mucosal lymphoid tissues and are notably, in the context of the immune-mediated lymphocytic cholangitis characteristic of PBC, involved in the localization of Th17 cells and CD8 effector T cells to cholangiocytes and the periductal area in portal tracts^16^. This study also reinforces the importance of IL-12 and JAK-STAT signalling in this disease.

The functional annotation of risk loci has helped to assign priority to the candidate genes at newly identified and established risk loci. Furthermore, the identification of disease-associated regulatory variants at multiple risk loci emphasizes the potential importance of gene regulation in the pathogenesis of PBC (and presumably other complex disorders). This possibility is corroborated by the finding of numerous risk loci wherein the index and/or closely related SNPs that appear to represent regulatory, mQTL and/or eQTLs variants related to the nearby gene. Via the FGWAS analysis, this study also suggests particular importance of CD20+ B cells and Th1 cells in the pathogenesis of PBC. However, both the cell types and the specific gene variants most relevant to PBC require further investigation and in particular exploration of the tissue-specific functional effects of the disease-associated variants.

By looking for drug–gene interactions, we have identified candidate drugs targeting specific, PBC-associated pathways, creating new opportunities to re-purpose available drugs for targeted immune therapy. Despite the speculative nature of this analysis, the data provide a start point in the search for novel therapies that are urgently needed to improve outcomes for PBC patients.

## Methods

### Study samples and genotyping

The use of human subjects for this study was approved by the University Health Network Research Ethics Board, The Mayo Clinic Institutional Review Board, Etico Indipendente IRCCS Istituto Clinico Humanitas, UC Davis Institutional Review Board and the Oxford Research Ethics Committee.

All PBC cases included in the Canadian–US, Italian and UK discovery and validation cohorts fulfilled the American Association for the Study of Liver Diseases criteria for PBC.

The Canadian–US discovery cohort included 499 PBC cases who were self-reported whites of European descent and 390 healthy Canadian controls, all genotyped using the Illumina HumanHap370 BeadChip. Additional controls included in this cohort were 1,094 control subjects provided from the Prostate Cancer Genetics Markers Susceptibility (CGEMS), 1,142 controls from the Breast CGEMS studies and 1,748 controls from the New York Cancer Project, all of whom who were genotyped on an Illumina 550 K bead array^4^. Following all quality control (QC) procedures, the final Canadian–US discovery set included 499 PBC cases and 4,374 controls.

The PBC cases included in the Italian discovery cohort were self-reported whites of Italian descent genotyped using the Illumina Human610-Quad BeadChip. Controls in this cohort were healthy Italians genotyped using the Illumina 1M-duo array. Following QC procedures, the final Italian discovery set comprised 449 cases and 940 controls.

The PBC cases included in the UK discovery cohort were self-reported whites of British descent, genotyped using the Illumina Human-660 W Quad array. Controls in this cohort were 5,163 population controls genotyped on the Illumina 1M-Duo array as part of the Wellcome Trust Case Control Consortium 2 project. Following QC procedures, the UK discovery set comprised 1,816 cases and 5,161 controls.

The ‘Canadian’ 903 PBC cases and 834 controls included in the validation studies were self-reported whites of European descent recruited from Canada, Europe and the United States to an ongoing PBC genetics study based in Toronto. The 721 ‘US’ PBC cases and 294 controls included in the validation studies were self-reported whites of European descent enroled in the Mayo Clinic PBC Genetic Epidemiology registry and biorepository based at the Mayo Clinic in Rochester (https://clinicaltrials.gov/ct2/show/NCT01161953?term=pbc&rank=5).The Italian PBC cases and controls included in the validation studies were self-reported whites of Italian descent recruited to the Italian PBC Genetics study based at Instituto Humanitas in Milan. The Italian controls were obtained from Ospedale Alessandro Manzoni, Lecco, Italy and were unrelated healthy volunteers with no known non-Italian heritage. Cases and controls from the Canadian, Italian and the US cohorts were genotyped at the University Health Network/Mount Sinai Hospital Clinical Genomics Centre using a Sequenom iPLEX Gold assay. Following QC procedures, the final validation set included 903 cases and 834 controls from Canada; 300 cases and 618 controls from Italy; and 721 cases and 294 controls from the United States (Supplementary Table 1).

The ‘UK’ PBC cases included in the validation studies were self-reported whites of British descent recruited to the UK-PBC project via the UK-PBC Consortium (http://www.uk-pbc.com/). Cases were genotyped using Sequenom iPLEX Gold assay at the Wellcome Trust Sanger Institute Genotyping Facility (http://www.sanger.ac.uk/). The UK validation control data were obtained from the TwinsUK resource, an adult twin registry comprising 12,000 (predominantly female) British twins. Genotype data for 3,512 twin individuals (genotyped using the Illumina HumanHap610 array) were obtained from the Department of Twin Research and Genetic Epidemiology at King’s College London. One twin from each genotyped pair was included in the current study, amounting to 2,603 unrelated individuals. Following QC procedures, the final UK validation set comprised 1,792 PBC cases and 2,515 TwinsUK controls (Supplementary Table 1).

### Quality control

We implemented a standard QC pipeline across all three discovery data sets, over-and-above QC procedures carried out in the respective primary analyses^2^,^4^,^5^. QC checks were carried out using the software package PLINK^17^. Within each discovery data set we removed SNPs with a genotype call rate <95%; minor allele frequency <0.05; significant deviation from Hardy Weinberg Equilibrium in controls (*P*<10^−5^) or a large difference (>5%) in the proportion of missing genotypes in cases versus controls. We removed samples showing high rates of missing data (>90%); whole-genome heterozygosity >six s.d. from the mean; estimated proportion of identity by descent (IBD) sharing with another sample >0.1, or apparent gender discrepancies (based on X-chromosomal heterozygosity >0.2 for men and <0.2 for women). Principal component analysis (based on a subset of 32,000 highly informative SNPs) was carried out using the ‘smartpca’ routine of the EIGENSOFT package^18^ to identify population outliers for exclusion and to identify principal components that differed between cases and controls; these principal components were used as covariates in subsequent association analyses.

### Genome-wide imputation

We used the SNPs and samples passing QC to carry out genome-wide imputation within each of our cohorts using the software package MaCH^19^ with HapMap3 CEU+TSI samples as reference data sets. Within each cohort we used approximately the same set of genotyped SNPs in cases and controls to ensure similar levels of informativity. Following imputation, we retained only those SNPs displaying minor allele frequency >0.005 and imputation quality score *R*^2^>0.5 in all three cohorts.

### Statistical analysis of discovery cohorts

Within each cohort we carried out association analysis of the genome-wide imputed data allowing for imputation uncertainty using the software package ProbABEL^20^. We performed logistic regression of disease phenotype on allele dosage; principal components that differed between cases and controls were included as covariates to help correct for population stratification. Quantile–quantile plots of the genome-wide set of test statistics were examined and genomic control correction was carried out within each cohort by multiplying the standard error of the estimated log odds ratio for each SNP by the square root of the genomic control inflation factor *λ* (ref. 21). The resulting log odds ratios and adjusted standard errors from all three cohorts were meta-analysed using the software package META to produce the final set of genome-wide discovery results^22^.

### Validation analysis

We selected loci for validation if they achieved suggestive level of significance in the discovery analysis (minimum *P*<2 × 10^−5^) and were not already known to be associated with PBC. We also selected loci for validation if they had achieved genome-wide significant association in one previous study but had never been validated in an independent cohort. We selected approximately two validation SNPs per locus; for loci displaying extended patterns of linkage disequilibrium or harbouring several putative independent association signals we attempted to select two validation SNPs within each subregion.

Within each locus chosen for validation we assigned priority to SNPs according to whether they had been genotyped in the TwinsUK cohort (which was used as a validation cohort for the UK validation cases). One SNP selected for validation (rs2297067) did not have genotype data available in TwinsUK and was therefore imputed within TwinsUK based on genotyped SNPs in the surrounding 5-Mb region using the software packages SHAPEIT^23^ and IMPUTE2 (ref. 24), with 1,000 Genomes (Phase I version 3 integrated data, released on March 2012) used as a reference sample. The TwinsUK cohort was subjected to a variety of additional QC checks as described previously^25^; the 2,515 controls used here correspond to the 2,520 controls used previously with an additional five exclusions due to discrepant gender^25^.

Within each validation cohort we carried out case/control association analysis of those SNPs that were successfully genotyped using logistic regression in PLINK. Results from the four validation cohorts (or from the combined discovery and validation cohorts) were combined via meta-analysis in META.

### Imputation to 1,000 Genomes within validated loci

Imputation within the discovery cohorts was carried out at the six validated loci using the software packages SHAPEIT^23^ and IMPUTE2 (ref. 24) with the 1,000 Genomes (Phase I integrated variant set, release December and June 2013) used as a reference panel. The same genotyped SNPs that had been used to inform HapMap3 imputation for the discovery analysis were used for the 1,000 Genomes imputation within these targeted regions. Association analysis of SNPs passing post-imputation QC (‘info’ score >0.5) was carried out separately within each cohort, the results were genomic control corrected by multiplying the standard error of the estimated log odds ratio for each SNP by the square root of the previously estimated genomic control inflation factor *λ* for each cohort, and results were combined across the cohorts via meta-analysis in META. This confirmed the findings from our original (HapMap3) imputation experiment but did not identify any substantially stronger associations or candidate causal variants than we had already found.

### Functional annotation of validated loci

Left and right boundaries for each associated region were defined by finding a 0.1-cM interval either side of the most strongly associated SNP where no SNP has *P*<1 × 10^−5^. We looked for overlap between PBC risk loci and confirmed risk loci for other autoimmune conditions using ImmunoBase, a web-based resource focused on the genetics and genomics of immunologically related human diseases (http://www.immunobase.org/). To assign priority to candidate genes and candidate variants at risk loci, we used the online PICS (Probabilistic Identification of Causal SNPs) algorithm to identify candidate variants at each risk locus with a PICS probability >0.0275 (http://www.broadinstitute.org/pubs/finemapping/?q=pics)^9^. We adopted this threshold to be consistent with Farh *et al*.^9^ in their paper describing the approach. Given an index SNP corresponding to the most associated SNP in a locus, the PICS algorithm calculates (based on the known linkage disequilibrium pattern in the region, as measured in a large Immunochip or 1000 Genomes reference sample) a score for each SNP in the region, representing the extent to which that SNP could, in fact, be the true causal SNP, allowing for statistical sampling variation.

We then used the Ensembl Variant Effect Predictor web tool to annotate candidate variants for their predicted functional consequences (http://www.ensembl.org/info/docs/tools/vep/index.html). We used Genevar to evaluate the measured effects of these variants on DNA methylation in tissue collected from 856 healthy female twins of the MuTHER resource (http://www.sanger.ac.uk/resources/software/genevar/)^26^,^27^. We used Genevar^26^, seeQTL (http://www.bios.unc.edu/research/genomic_software/seeQTL/)^28^ and the University of Chicago eQTL browser (http://eqtl.uchicago.edu/cgi-bin/gbrowse/eqtl/) to identify eQTLs amongst candidate variants.

We also used the FGWAS software and its associated annotation file (containing 450 genomic annotations of various types), applied to our full set of GWMA results, to investigate the extent to which genetic variants associated with PBC were enriched within specific annotation categories^12^. Testing each annotation individually, we found 75 annotations that showed enrichment (*P*<0.01) of GWMA association signals; as many of these annotations are correlated with one another we used a stepwise selection approach followed by cross-validation to mitigate overfitting (similar to the procedure performed by Pickrell^12^) on these 75 annotations to identify a final best-fitting model that included 6 annotations. Annotation information used by FGWAS was derived from a variety of sources including Maurano *et al*.^29^, Thurman *et al*.^30^ and Hffman *et al*.^31^ (see Appendix of Pickrell^12^ for details).

### Pathway analysis

Using summary results from the GWMA (effect size, standard error and allele frequency) along with SNP linkage disequilibrium estimated from the Italian GWAS individual-level genotype data, we performed approximate conditional analysis using the software GCTA^13^. Only the independently associated signals with conditional *P* value and *P*_GWMA_ both <0.001 were retained for further consideration. We submitted the rsIDs and *P*_GWMA_ of these SNPs as well as gene sets from BioCarta, KEGG, PID and Reactome curated by MSigDB (as of 26 March 2014) to the i-GSEA4GWAS web server^14^. This programme identified genes within 20 kb of the SNPs and represented each gene by the greatest –log *P*_GWMA_ of the SNP(s) mapped to it. Gene sets were then assessed for enrichment with significant genes while SNP label permutations were conducted to correct for bias from variations in gene size and gene set size. False discovery rate was used to correct for multiple testing based on the distributions of enrichment scores generated by permutation.

### Drug-pathway analysis

To identify drugs that affected the pathways associated with PBC (when the HLA locus was excluded), we first identified the genes participating in each pathway. We then downloaded drug–gene associations from the Drug Gene Interaction database^15^ and scored each drug by the proportion of each its targets that were in each pathway, which we termed as the drug’s pathway specificity. As a secondary scoring metric, we evaluated the proportion of each pathway affected by the drug using the Jaccard index on the respective sets of pathway genes and targeted genes. To identify promising drug candidates, we ranked drugs first by our primary specificity metric and then by the secondary Jaccard index.

## Additional information

**How to cite this article:** Cordell, H. J. *et al*. International genome-wide meta-analysis identifies new primary biliary cirrhosis risk loci and targetable pathogenic pathways. *Nat. Commun*. 6:8019 doi: 10.1038/ncomms9019 (2015).

## Supplementary Material

Supplementary Information Supplementary Figures 1-5, Supplementary Tables 1-4 and Supplementary References

Supplementary Data 1 SNPs taken forward for genotyping in the validation cohort

Supplementary Data 2 Most-likely causal variants (‘candidate variants’) at PBC risk loci

Supplementary Data 3 Regulatory feature variants at PBC risk loci

Supplementary Data 4 Methylation quantitative trait loci (mQTLs) at PBC risk loci

Supplementary Data 5 Expression quantitative trait loci (eQTLs) at PBC risk loci

Supplementary Data 6 Enrichment of genomic annotations in FGWAS

Supplementary Data 7 Jaccard Index and Specificity Score of drugs for PBC-associated gene sets

## Figures and Tables

**Table 1:** PBC risk loci identified in the current study.

**a. Confirmed risk loci (validation** ***P*****<4.4 × 10**^**−4**^ **resulting in combined** ***P*****<5 × 10**^**−8**^)										
**Locus**	**SNP**	**Position (build 38)**	**A1/A2**	**Discovery** ***P***	**Validation** ***P***	**Joint** ***P***	**OR (95% CI)**	**Region (build 38)**	**Nearby genes and functional annotation** *	**Autoimmune overlap**
2q12.1	rs12712133	102,249,813	A/G	1.62 × 10^**−**5^	7.94 × 10^**−**5^	5.19 × 10^**−**9^	1.14 (1.07–1.21)	102,118,975–102,438,307	*IL1R1*, *IL1RL2*†, *FAM183DP*, *IL1RL1*‡, *IL18R1*, *LOC100422339*, *IL18RAP*, *MIR4772*	CD, CeD
2q36.3	rs4973341	227,795,646	C/T	6.48 × 10^**−**7^	7.73 × 10^**−**5^	2.34 × 10^**−**10^	0.82 (0.74–0.90)	227,747,828–227,815,647	*RNA5SP121*, *SNRPGP8*, *LOC100533842*, *CCL20*†^,^§	
4p16.3	rs11724804	971,991	A/G	3.67 × 10^**−**7^	4.25 × 10^**−**6^	9.01 × 10^**−**12^	1.22 (1.12–1.33)	853,681–1,014,424	*GAK*, *TMEM175*, *DGKQ*†, *SLC26A1*a, *IDUA*, *FGFRL1*	
5q21.1	rs526231	103,345,680	T/C	3.10 × 10^**−**5^	9.39 × 10^**−**5^	1.14 × 10^**−**8^	0.87 (0.81–0.93)	102,939,698–103,416,571	*PAM*§, *EIF3KP1*, *GIN1*, *PPIP5K2*, *C5orf30*‡^,^§	RA
5q33.3	rs2546890	159,332,892	G/A	1.20 × 10^**−**6^	1.89 × 10^**−**5^	1.06 × 10^**−**10^	0.87 (0.82–0.93)	159,117,927–159,414,310	*RNF145*, *UBLCP1*, *RNU4ATAC2P*, *IL12B*, *LOC285626*†	Pso, MS, CD
6q23.3	rs6933404	137,638,098	C/T	9.47 × 10^**−**7^	2.84 × 10^**−**5^	1.27 × 10^**−**10^	1.18 (1.09–1.27)	137,571,557–137,803,754	*LOC102723649*, *LOC442263*, *OLIG3*†, *TNFAIP3*†	RA, SLE, SjS, CeD, UC, MS

**b. Suggestive risk loci (validation** ***P*****<1 × 10**^**−3**^)										
**Locus**	**SNP**	**Position (build 38)**	**A1/A2**	**Discovery** ***P***	**Validation** ***P***	**Joint** ***P***	**OR for A1 (95% CI)**	**Region (build 38)**	**Nearby genes and functional annotation** *	**Autoimmune overlap**
5q23.1	rs2434360	116,057,393	T/G	3.20 × 10^**−**3^	9.94 × 10^**−**4^	1.04 × 10^**−**5^	1.14 (1.05–1.23)	116,032,882–116,163,459	*RPS25P6*, *ARL14EPL*, *COMMD10*	
16p11	rs1859308	27,386,677	T/C	7.72 × 10^**−**5^	5.37 × 10^**−**4^	1.63 × 10^**−**7^	0.85 (0.77–0.93)	27,359,133–27,434,733	*IL4R*, *IL21R*	

A1, tested allele; CD, Crohn disease; CeD, coeliac disease; CI, confidence interval; MS, multiple sclerosis; OR, odds ratio in validation cohorts; Pso, psoriasis; RA, rheumatoid arthritis; SjS, Sjogren syndrome; SLE, systemic lupus erythematosus; SNP, single-nucleotide polymorphism; UC, ulcerative colitis.

PBC risk loci identified in the current study. SNPs were taken forward for validation based on having a discovery *P* value <2 × 10^**−**5^ (or, in the case of rs526231 and rs2434360, based on acting as a proxy for a SNP with a *P* value <2 × 10^**−**5^). Discovery *P* values were calculated using logistic regression of individual discovery data sets in ProbABEL followed by genomic control correction of individual discovery data sets in R and fixed-effects meta-analysis in META; validation *P* values were calculated using logistic regression of individual data sets in PLINK followed by fixed-effect meta-analysis in META; joint *P* values were calculated using fixed-effect meta-analysis of discovery and validation data sets in META; see Methods. Autoimmune overlap refers to overlap between risk loci for PBC and those of other autoimmune conditions.

^*^Functional annotation.

^†^Regulatory variants: The index SNP or variants in strong linkage disequilibrium (LD, *r*^2^≥0.8) with the index SNP at this locus overlap regulatory elements that are related to the annotated gene (Supplementary Table 3).

^‡^mQTLs: The index SNP or variants in strong LD are correlated to methylation related to the annotated gene (Supplementary Data 4).

^§^eQTLs: The index SNP or variants in strong LD are correlated to expression of the annotated gene (see Supplementary Data 3).

**Table 2:** Results from pathway analysis in iGSEA4GWAS.

**Gene set**	**Source**	**FDR (with HLA)**	**FDR (without HLA)**
NO2-dependent IL-12 pathway in NK cells	Biocarta	6.7 × 10^−4^	
JAK-STAT signalling pathway*^,^†	KEGG	0.001	0.013
IL-12 mediated signalling events	PID	0.001	
IL-12- and Stat4-dependent signalling in Th1 development*^,^†	Biocarta		<0.001
Interferon signalling	REACTOME	0.001	
PD-1 signalling	REACTOME	0.001	
Phosphorylation of CD3 and TCR-ζ chains	REACTOME	0.001	
IL-27-mediated signalling events*^,^†	PID	0.001	<0.001
Cytokine–cytokine receptor interaction‡^,^§^,^||^,^¶^,^#^,^**^,^†^,^††^,^‡‡	KEGG	0.002	0.010
IFN-γ signalling	REACTOME	0.002	
MHC class II antigen presentation	REACTOME	0.003	
Cytokine signalling in immune system	REACTOME	0.004	
Antigen processing and presentation	KEGG	0.004	
Intestinal immune network for IgA production	KEGG	0.004	
Co-stimulation by the CD28 family	REACTOME	0.005	
IL-2 mediated signalling events	PID	0.008	
TCR signalling	REACTOME	0.008	
Downstream TCR signalling	REACTOME	0.008	
Cell adhesion molecules	KEGG	0.015	
Th1, Th2 differentiation†	Biocarta	0.019	0.012
IL-2 receptor beta chain in T-cell activation	Biocarta	0.021	
Interferon α/β signalling	REACTOME	0.035	
IL-23-mediated signalling events	PID	0.039	

FDR, false discovery rate; IFN, interferon; IL, interleukin; PD, programmed cell death; TCR, T cell antigen receptor; NK, natural killer.

Gene sets with FDR <0.05 are listed. Results are shown with or without inclusion in the analysis of SNPs within the HLA region. The top 10 hits from our drug-positioning analysis using a combined pathway from the HLA excluded set are indicated by symbols for the associated pathways that they affect.

^*^Tofacitinib.

^†^Glatiramer acetate.

^‡^Axitinib.

^§^Pazopanib.

^||^Vatalanib.

^¶^Cediranib.

^#^X-82.

^**^Telatinib.

^††^Linifanib.

^‡‡^Tandutinib.
