# Supplementary material for: International genome-wide meta-analysis identifies new primary biliary cirrhosis risk loci and targetable pathogenic pathways
Source: Nat Commun. 2015 Sep 22;6:8019. doi: 10.1038/ncomms9019 (PMC4580981; doi:10.1038/ncomms9019)
Supplement: Supplementary Information — Supplementary Figures 1-5, Supplementary Tables 1-4 and Supplementary References [file ncomms9019-s1.pdf]

Supplementary Figure 1: QQ plot after GC correction

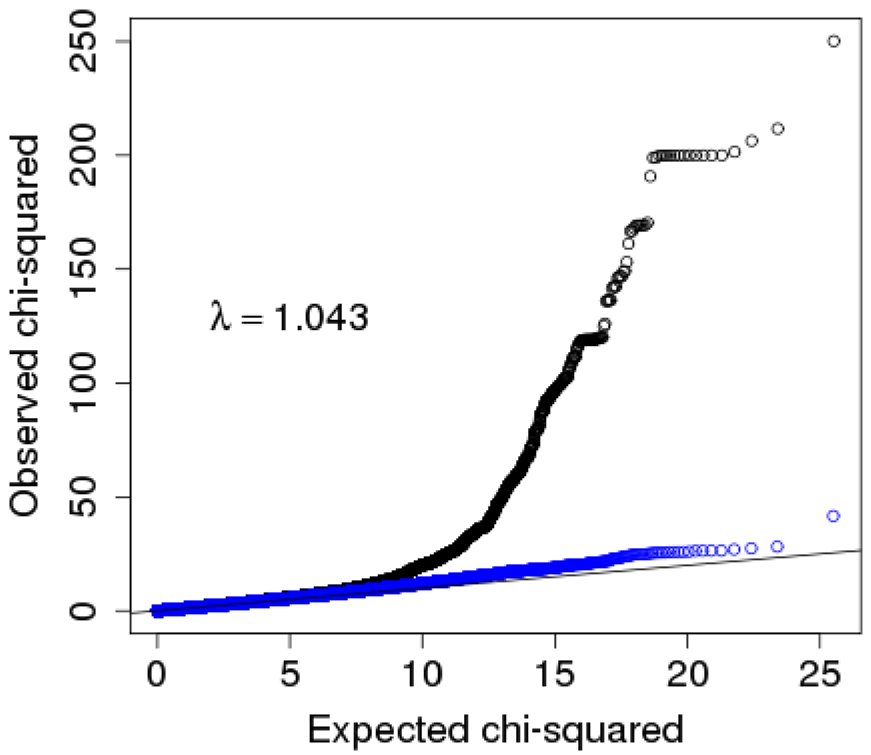

Quantile-Quantile (QQ) plots of GWMA results. Black circles indicate results seen at full set of genotyped and imputed SNPs. Blue circles indicate results seen after removal of SNPs in previously identified PBC-associated loci. After genomic control (GC) correction in each discovery cohort individually, the inflation factor was λ = 1.057 for all SNPs and λ = 1.043 with SNPs at established risk loci excluded, indicating no substantial departure of test statistics from their expected distribution. Note that even with established risk loci excluded, there is still deviation in the tail of the distribution, suggesting the presence of true associations within the dataset.

Supplementary Figure 2: Manhattan plots of GWMA results

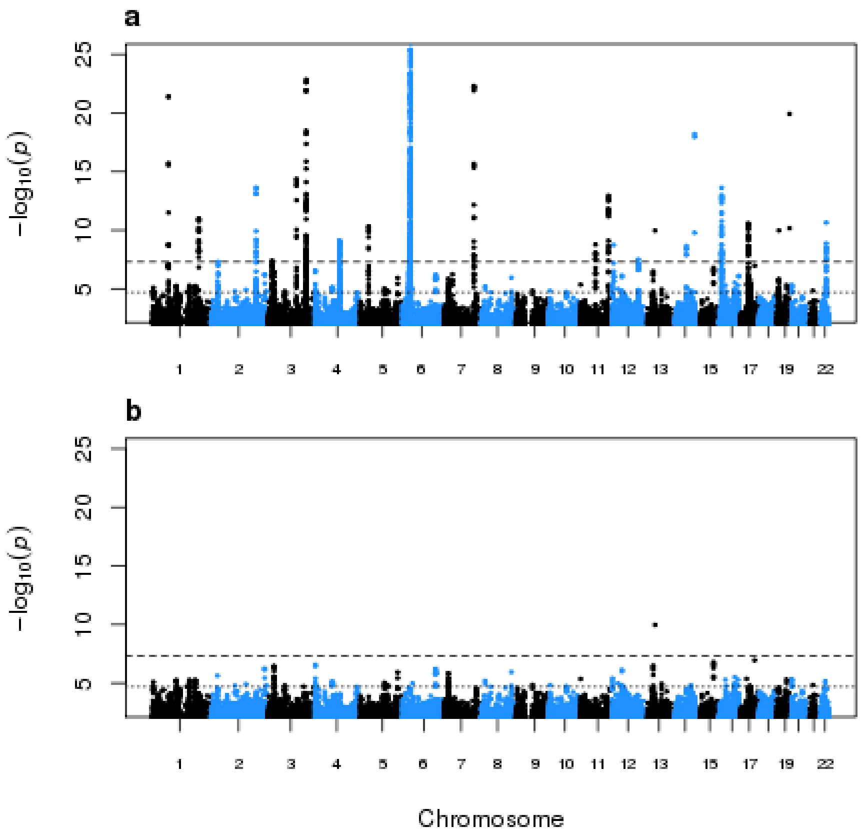

Manhattan plots of GWMA results. Dashed lines indicate genome-wide significance threshold (P=5×10^{-8}). Dotted lines indicate suggestive significance threshold (P=2×10^{-5}). (a) Association signals seen at full set of genotyped and imputed SNPs. (b) Association signals seen after removal of SNPs in previously identified PBC associated loci.

Supplementary Figure 3: LocusZoom plots of the six newly-identified PBC loci

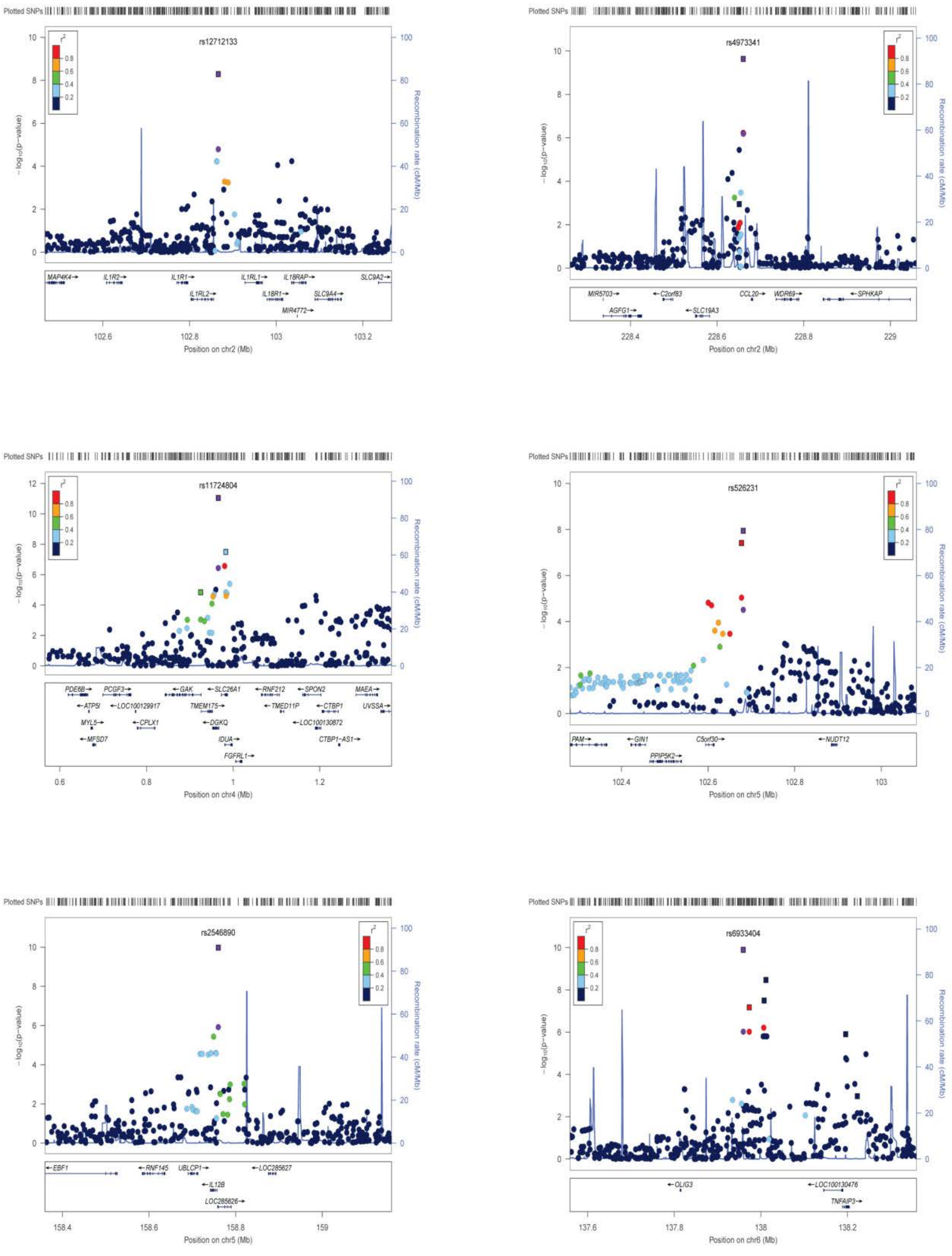

LocusZoom plots of the six newly-identified PBC loci. Genes and ESTs within the region are shown in the lower panels. The unbroken blue line indicates the recombination rate within the region. Each filled circle represents the P value for one SNP in the discovery cohort, with the top SNP listed shown in purple and other SNPs in the region colored depending on their expected degree of correlation (r^2) with the top SNP (as estimated internally by LocusZoom on the basis of 1000 Genomes European haplotypes from March 2012). The P values for the SNPs followed up in each region when analyzed in the combined discovery and validation cohorts are shown as filled squares.

Supplementary Figure 4: Forest plots for the six validating loci

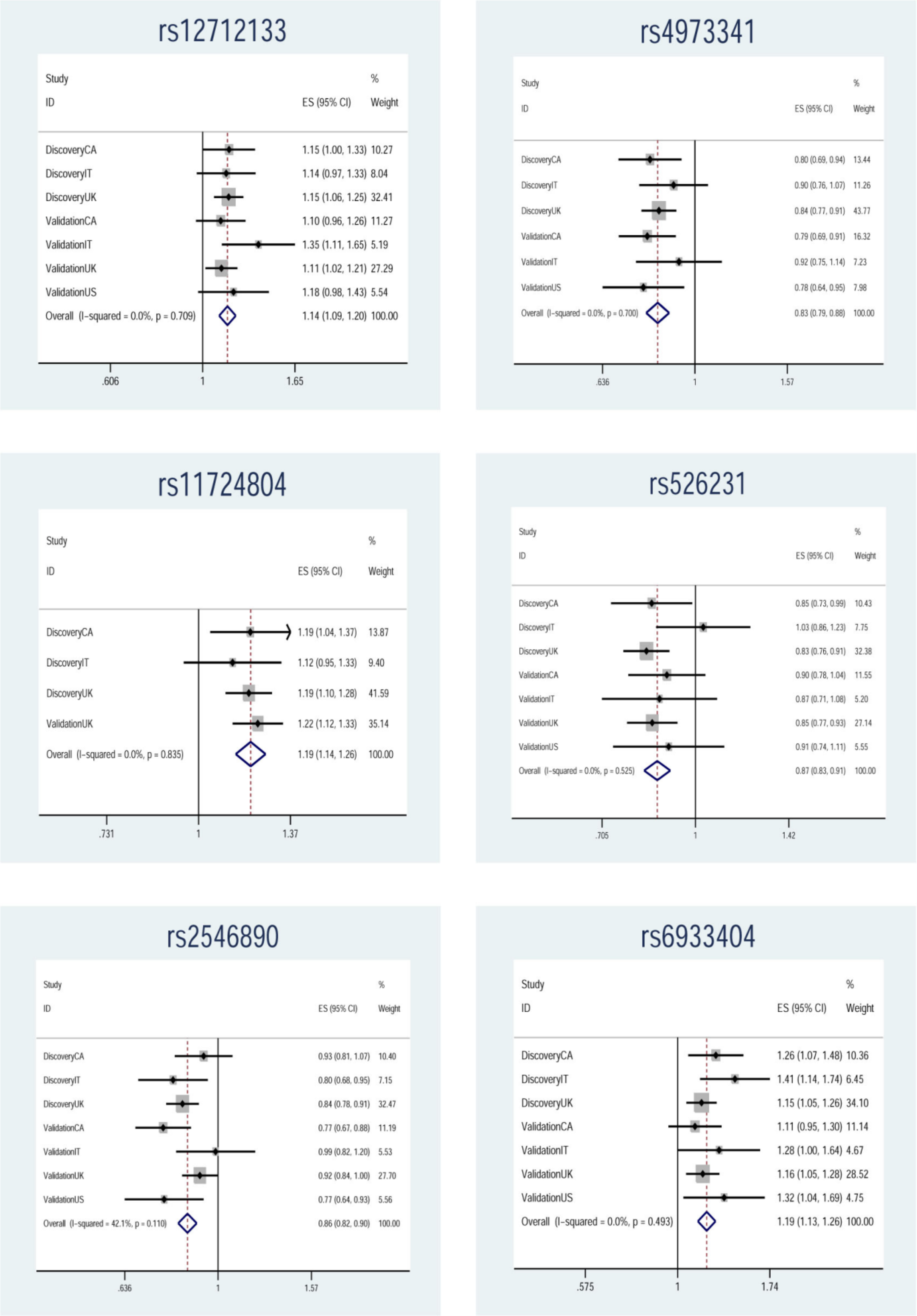

Forest plots for the six validating loci. Shown are odds ratio estimates (ES) and 95% confidence intervals (CI; as indicated by horizontal lines) from an inverse-variance weighted fixed-effect meta-analysis of discovery and validation cohorts for each of the six newly-identified PBC loci. CA: Canadian cohort; IT: Italian cohort; UK: UK cohort; US: US cohort.

Supplementary Figure 5: (a) Forest plots and (b) LocusZoom plots for the two suggestive newly-identified PBC loci

a

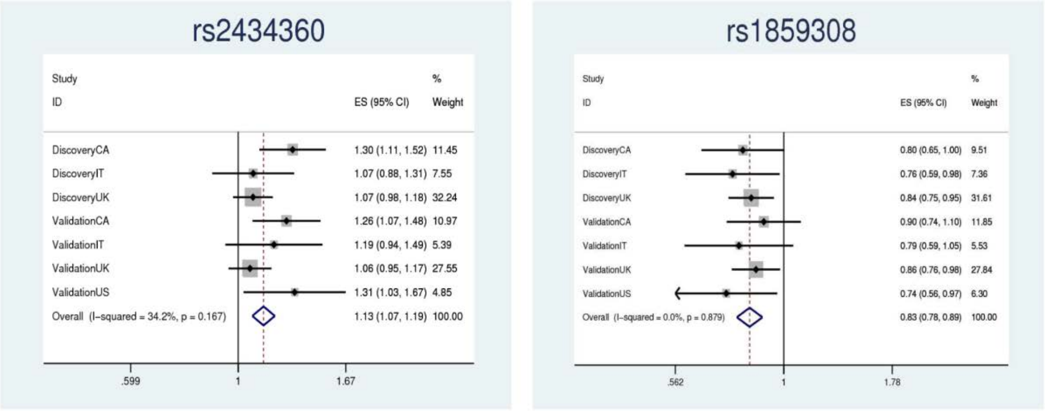

b

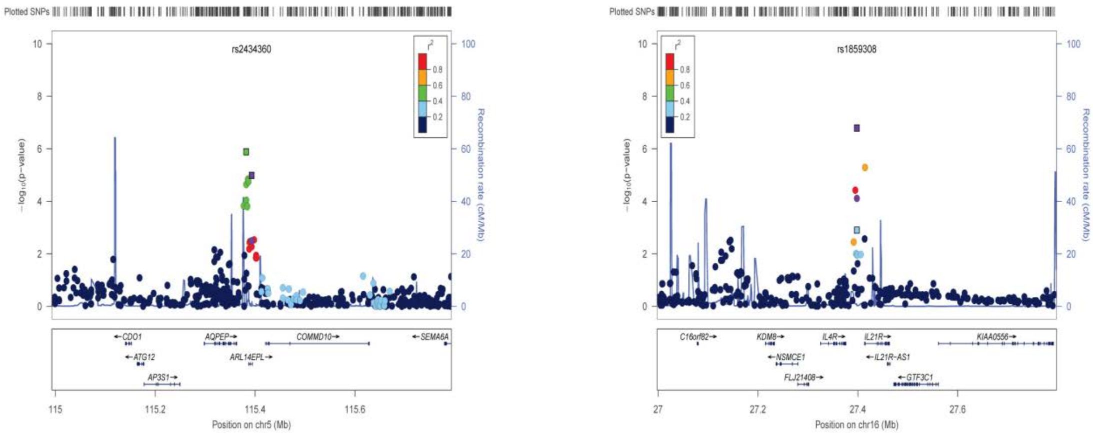

(a) Forest plots and (b) LocusZoom plots for the two suggestive newly-identified PBC loci. See legends to Supplementary Figures S3 and S4 for full description.

Supplementary Table 1: Sample size

Country	Discovery		Validation	
	Cases	Controls	Cases	Controls
Canada	499	4,374	903	834
Italy	449	940	300	618
United Kingdom	1,816	5,161	1,792	2,515
United States of America	-	-	721	294
**Total**	**2,764**	**10,475**	**3,716**	**4,261**

The sample size used in the discovery and validation cohorts in the current study. Discovery cohorts corresponded to data sets used in previously published North American, Italian and UK GWAS of PBC.[1-3] Validation cohorts consisted of newly collected cases together with (new or existing) independent controls.

Supplementary Table 2: Loci achieving genome-wide level of significance in the discovery analysis

Locus	SNP	A1/A2	*ƒ*(A1)	*P*	OR	Candidate Gene/s and functional annotation	Autoimmune overlap	Known
1p31.3	rs6679356	C/T	0.21	7.49×10^{-28}	1.52	*IL12RB2* , *SERBP1* †		Yes[1-6]
1q31.3	rs17641524	C/T	0.83	1.01×10^{-11}	0.77	*DENND1B* *†‡	CD, UC	Yes[3,5,6]
2p23.1	rs4952108	C/T	0.81	5.05×10^{-8}	1.28	*LBH* *	RA	Yes[3]
2q32.3	rs3771317	T/C	0.86	2.32×10^{-14}	0.71	*NAB1* *†, *STAT1* , *STAT4*		Yes[1,3,5,6]
3p24.3	rs1372072	A/G	0.38	3.99×10^{-8}	1.20	*PLCL2* *†ϕ	RA	Yes[3,5,6]
3q13.33	rs2293370	G/A	0.86	4.26×10^{-15}	1.42	*TMEM39A* †, *CD80*	Vitiligo, MS, CeD	Yes[3,5,6]
3q25.33	rs485499	C/T	0.40	1.43×10^{-23}	0.71	*IL12A* †ϕ, *SCHIP1* ϕ	SSc, SjS	Yes[1-6]
4q24	rs1054037	T/C	0.52	8.31×10^{-10}	1.22	*MANBA* ϕ, *NFKB1* , *CISD2* ϕ	UC, MS	Yes[3,5,6]
5p13.2	rs860413	A/C	0.79	4.73×10^{-11}	1.28	*IL7R* , *CAPSL* †, *UGT3A1* †	MS, UC	Yes[3,5,6]
6p21.3	rs7774434	C/T	0.47	2.37×10^{-56}	1.68	Many (MHC)	Many	Yes[1-6]
7q32.1	rs10488631	T/C	0.91	5.08×10^{-23}	0.63	*IRF5* †ϕ, *TNP03* ϕ	SSc, SjS, RA, UC, SLE	Yes[1-6]
11q13.1	rs510372	T/C	0.43	1.68×10^{-9}	0.81	*CCDC88B* *†ϕ, others	Alopecia, CD, UC, MS	Yes[3,5,6]
11q23.3	rs6421571	C/T	0.80	1.78×10^{-13}	1.39	*CXCR5* *, *DDX6*	RA, CD, SjS, CeD, MS	Yes[3,5,6]
12p13.31	rs1800693	T/C	0.49	1.84×10^{-9}	0.82	*TNFRSF1A* *†, *PLEKHG6* †	Spondylitis, MS	Yes[3,5,6]
12q24.12	rs11065987	A/G	0.63	3.20×10^{-8}	0.84	*ATXN2* †, *SH2B3* , *TRAFD1* ϕ, *ALDH2* ϕ	PSC, JIA, Spondylitis, RA, T1D, Vitiligo, CeD	Yes[6]
13q14.3	rs9591325	T/C	0.94	1.07×10^{-10}	1.63	*DLEU1* *		No
14q24.1	rs911263	T/C	0.73	2.25×10^{-9}	1.24	*RAD51L1* †, *RAD51B* *	RA	Yes[3,5,6]
14q32.32	rs2297067	C/T	0.82	6.34×10^{-19}	0.72	*EXOC3L4* *†ɣ, *TNFAIP2*		Yes[3]
16p13.13	rs12924729	G/A	0.67	2.39×10^{-14}	1.31	*SOCS1* , *CLEC16A* *†, *DEXI* ϕ	MS, T1D	Yes[3,5,6]
17q12	rs9303277	C/T	0.50	2.57×10^{-11}	0.81	*IKZF3* *†ϕ, others	CD, RA, MS, T1D, UC	Yes[1-6]
19p13.2	rs2304256	A/C	0.29	1.05×10^{-10}	0.79	*TYK2* ϕɣ	RA, MS, T1D, CD, JIA, UC, Pso, Spondylitis	Yes[6]
19q13.3	rs3745516	G/A	0.76	1.22×10^{-20}	0.72	*SPIB* ɣ, *MYBPC2* †		Yes[1-4]
22q13.1	rs2069235	G/A	0.68	2.23×10^{-11}	0.79	*SYNGR1* *†ϕ, *PDGFB* ϕ	UC, RA, CD	Yes[3,5,6]

Discovery P-values were calculated using logistic regression of individual discovery datasets in ProbABEL followed by genomic control correction of individual discovery datasets in R and fixed-effects meta-analysis in META. Known indicates whether the risk locus has been identified in a previous study (with references). Note that 2p23.1 was implicated in Mells *et al* (2011) but failed to replicate. Note also that 13q14.3 has not been identified in previous studies but it is probably a spurious finding. SNP, single nucleotide polymorphism; A1, tested allele; *ƒ*(A1), frequency of the tested allele; OR, odds ratio; CD, Crohn disease; UC, ulcerative colitis; RA, rheumatoid arthritis; MS, multiple sclerosis; CeD, celiac disease; SSc, systemic sclerosis; SjS, Sjogren syndrome; SLE, systemic lupus erythematosus; PSC, primary sclerosing cholangitis; JIA, juvenile inflammatory arthritis; T1D, type 1 diabetes mellitus; Pso, psoriasis.

Functional Annotation

*Regulatory region variants: the candidate variant is located in a regulatory region in proximity to the annotated gene (Supplementary Table S6); †Methylation quantitative trait loci (mQTLs): the candidate variant is correlated to methylation related to the annotated gene (Supplementary Table S7); ϕExpression quantitative trait loci (eQTLs): the candidate variant is correlated to expression of the annotated gene (see Supplementary Table S8); ‡Splice region variants: the candidate variant is predicted to alter slicing of the annotated gene (see Supplementary Table S5); ɣMissense variants: the candidate variant causes a non-synonymous change in the coding sequence of the annotated gene (Supplementary Table S5).

Supplementary Table 3: Splice region and missense variants at PBC risk loci

a. Splice region variants

Locus	SNP	Position (build 38)	A1	*f* (A1)	LD (r^2)	Gene
1q31.3	rs17641524	01:197735587	T	T:0.18	Index	*DENND1B*

b. Missense variants

Locus	SNP	Position (build 38)	A1	*f* (A1)	LD (r^2)	Gene	Codons	Amino acids	SIFT (score)	PolyPhen (score)
14q32.32	rs2297067	14:103100448	T	T:0.22	Index	*EXOC3L4*	Cgg/Tgg	R/W	deleterious (0)	probably damaging (0.942)
14q32.32	rs2297066	14:103100498	G	G:0.22	1	*EXOC3L4*	gaC/gaG	D/E	tolerated (1)	benign (0)
14q32.32	rs10131298	14:103102277	A	A:0.22	0.96	*EXOC3L4*	cTt/cAt	L/H	tolerated (0.13)	benign (0.005)
19p13.2	rs2304256	19:10364976	A	A:0.28	Index	*TYK2*	Gtc/Ttc	V/F	deleterious (0.05)	benign (0.042)
19q13.3	rs11546996	19:50423008	C	C:0.79	1	*SPIB*	Gca/Cca	A/P	tolerated (0.36)	benign (0.188)

Candidate variants at PBC risk loci that are splice region variants or missense variants. These transcript variants were identified using the Variant Effect Prediction (VEP) application in Ensembl. SNP, single nucleotide polymorphism; A1, minor allele; *f* (A1), minor allele frequency in CEU population.

Supplementary Table 4: Enrichment of genomic annotations in FGWAS after step-wise selection

Annotation Type	Annotation details/cell type	Source	ln(enrichment) parameter (95%CI)	*P*
Region of repressed chromatin	Lymphoblastoid cell line	Hoffman et al. (2013) (ENCODE)	-3.55 (<-20, -2.12)	1.71E-14
DNase-I hypersensitivity	Lymphoblastoid cell line	Maurano et al. (2012)	1.45 (0.69, 2.18)	4.13E-06
DNase-I hypersensitivity	CD20+ cells	Maurano et al. (2012)	1.69 (0.92, 2.46)	0.0360
DNase-I hypersensitivity	Promyelocytic leukaemia	Maurano et al. (2012)	-1.88 (-3.81, -0.67)	0.0031
DNase-I hypersensitivity	Th1 T cells	Thurman et al. (2012) (ENCODE)	1.88 (1.00, 2.65)	0.0063
DNase-I hypersensitivity	K562 leukaemia cell line	Maurano et al. (2012)	-1.98 (-4.93, -0.44)	0.0150

Listed are the final set of 6 enriched genomic annotations chosen by stepwise selection followed by cross-validation (to mitigate overfitting) from the 75 annotations that showed individual enrichment (P<0.01) of GWMA association signals according to FGWAS.

Supplementary References

1. Hirschfield, G.M. et al. Primary biliary cirrhosis associated with HLA, IL12A, and IL12RB2 variants. *N Engl J Med* **360**, 2544-55 (2009).
2. Liu, X. et al. Genome-wide meta-analyses identify three loci associated with primary biliary cirrhosis. *Nat Genet* **42**, 658-60 (2010).
3. Mells, G.F. et al. Genome-wide association study identifies 12 new susceptibility loci for primary biliary cirrhosis. *Nat Genet* **43**, 329-32 (2011).
4. Hirschfield, G.M. et al. Variants at IRF5-TNPO3, 17q12-21 and MMEL1 are associated with primary biliary cirrhosis. *Nat Genet* **42**, 655-7 (2010).
5. Juran, B.D. et al. Immunochip analyses identify a novel risk locus for primary biliary cirrhosis at 13q14, multiple independent associations at four established risk loci and epistasis between 1p31 and 7q32 risk variants. *Hum Mol Genet* **21**, 5209-21 (2012).
6. Liu, J.Z. et al. Dense fine-mapping study identifies new susceptibility loci for primary biliary cirrhosis. *Nat Genet* **44**, 1137-41 (2012).
7. Grundberg, E. et al. Global analysis of DNA methylation variation in adipose tissue from twins reveals links to disease-associated variants in distal regulatory elements. *Am J Hum Genet* **93**, 876-90 (2013).
8. Xia, K. *et al.* seeQTL: a searchable database for human eQTLs. *Bioinformatics* **28**, 451-2 (2012).
9. Yang, T.P. *et al.* Genevar: a database and Java application for the analysis and visualization of SNP-gene associations in eQTL studies. *Bioinformatics* **26**, 2474-6 (2010).
10. Stranger, B.E. *et al*. Population genomics of human gene expression. *Nat Genet* **39**, 1217-24 (2007).
11. Dimas, A.S. *et al*. Common regulatory variation impacts gene expression in a cell type-dependent manner. *Science* **325**, 1246-50 (2009).
12. Montgomery, S.B. *et al*. Transcriptome genetics using second generation sequencing in a Caucasian population. *Nature* **464**, 773-7 (2010).
13. Zeller, T. *et al*. Genetics and beyond--the transcriptome of human monocytes and disease susceptibility. *PLoS One* **5**, e10693 (2010).
14. Grundberg, E. *et al*. Mapping cis- and trans-regulatory effects across multiple tissues in twins. *Nat Genet* **44**, 1084-9 (2012).
15. Mangravite, L.M. *et al*. A statin-dependent QTL for GATM expression is associated with statin-induced myopathy. *Nature* **502**, 377-80 (2013).
16. Pickrell, J.K. Joint analysis of functional genomic data and genome-wide association studies of 18 human traits. *Am J Hum Genet* **94**, 559-73 (2014).
17. Maurano, M.T. et al. Systematic localization of common disease-associated variation in regulatory DNA. *Science* **337**, 1190-5 (2012).
18. Thurman, R.E. et al. The accessible chromatin landscape of the human genome. *Nature* **489**, 75-82 (2012).
19. Hoffman, M.M. et al. Integrative annotation of chromatin elements from ENCODE data. *Nucleic Acids Res* **41**, 827-41 (2013).
